# Capturing transient antibody conformations with DNA origami epitopes

**DOI:** 10.1038/s41467-020-16949-4

**Published:** 2020-06-19

**Authors:** Ping Zhang, Xiaoguo Liu, Pi Liu, Fei Wang, Hirotaka Ariyama, Toshio Ando, Jianping Lin, Lihua Wang, Jun Hu, Bin Li, Chunhai Fan

**Affiliations:** 1 CAS Key Laboratory of Interfacial Physics and Technology, Shanghai Institute of Applied Physics, Chinese Academy of Sciences, University of Chinese Academy of Sciences, Shanghai, 201800 China; 2 School of Chemistry and Chemical Engineering, Frontiers Science Center for Transformative Molecules, Institute of Translational Medicine, Shanghai, 200240 China; 3 State Key Laboratory of Medicinal Chemical Biology, College of Pharmacy and Tianjin Key Laboratory of Molecular Drug Research, Nankai University, Haihe Education Park, 38 Tongyan Road, Tianjin, 300353 China; 4 Biodesign Center, Tianjin Institute of Industrial Biotechnology, Chinese Academy of Sciences, Tianjin, 300308 China; 5 Joint Research Center for Precision Medicine, Shanghai Jiao Tong University Affiliated Sixth People’s Hospital South Campus, Southern Medical University Affiliated Fengxian Hospital, Shanghai, 201499 China; 6 Nano Life Science Institute (WPI NanoLSI), Kanazawa University, Kakuma-machi, Kanazawa, 920-1192 Japan; 7 Shanghai Synchrotron Radiation Center, Shanghai Advanced Research Institute, Chinese Academy of Sciences, Shanghai, 201210 China; 8 Shanghai Key Laboratory of Green Chemistry and Chemical Processes, School of Chemistry and Molecular Engineering, East China Normal University, 500 Dongchuan Road, Shanghai, 200241 China; 9 Institute of Molecular Medicine, Shanghai Key Laboratory for Nucleic Acids Chemistry and Nanomedicine, Renji Hospital, School of Medicine, Shanghai Jiao Tong University, Shanghai, 200127 China

**Keywords:** Self-assembly, Organizing materials with DNA

## Abstract

Revealing antibody-antigen interactions at the single-molecule level will deepen our understanding of immunology. However, structural determination under crystal or cryogenic conditions does not provide temporal resolution for resolving transient, physiologically or pathologically relevant functional antibody-antigen complexes. Here, we develop a triangular DNA origami framework with site-specifically anchored and spatially organized artificial epitopes to capture transient conformations of immunoglobulin Gs (IgGs) at room temperature. The DNA origami epitopes (DOEs) allows programmed spatial distribution of epitope spikes, which enables direct imaging of functional complexes with atomic force microscopy (AFM). We establish the critical dependence of the IgG avidity on the lateral distance of epitopes within 3–20 nm at the single-molecule level. High-speed AFM imaging of transient conformations further provides structural and dynamic evidence for the IgG avidity from monovalent to bivalent in a single event, which sheds light on various applications including virus neutralization, diagnostic detection and cancer immunotherapy.

## Abstract (web summary)

Understanding antibody-antigen interactions is important to deepen the understanding of immunology. Here, the authors report on the application of DNA origami structures for the controlled presentation of antigens to study antibody binding behaviours at room temperature.

## Introduction

Antibody (Ab)–antigen (Ag) interactions are important natural defense strategies in the mammalian immune system. Their unparalleled specificity has been popularly exploited for developing tools in clinical diagnosis, treatment, and prevention, as exemplified by conventional vaccine prevention and rapidly emerging cancer immunotherapy^1–7^. The fundamental mechanism of Ab–Ag-binding processes is of great importance for understanding immunology, however, revealing dynamic Ab–Ag interactions at the single-molecule level remains difficult^8^. Historically, the “Lock-and-Key” theory was proposed to explain the intrinsic capability of Abs to bind their cognate Ags with high specificity. Later on, the “induced-fit” model was evolved to describe Ab–Ag interactions by taking consideration of transient conformational changes of Abs/Ags^9,10^. As a powerful method to determine protein structures with the atomistic resolution, X-ray crystallography provides a route to experimentally examine modes of Ab–Ag interactions^11,12^. Cryogenic electron microscopy (cryo-EM) serves as a tool for imaging individual Abs at atomic resolution, offering unprecedented capability to determine complex structures. But important information on conformational flexibility and intermediate Ab–Ag complexes are likely lost due to the lack of temporal resolution ability of cryo-EM^13,14^.

Atomic force microscopy (AFM) has allowed the imaging of biomolecules under physiological conditions^15–19^, which provides an alternative route to probe Ab–Ag interactions^20–23^. Previous studies have demonstrated the potential of AFM for imaging Abs with near-atomistic resolution^24–26^ and its ability for quantitatively mapping nanomechanical forces of Ab–Ag interactions at the single-molecule level^27–29^. Recently, high-speed (HS) AFM offers up to video-rate temporal resolution, revealing certain types of dynamic processes of IgGs, e.g., walking on the viral surface, oligomerization, and complement activation upon antigen recognition^24,30,31^. These exciting advances in unveiling distinctive molecular events of Abs reveal its compelling implications in resolving heterogeneous conformations of Ab–Ag complexes. Nevertheless, the spatial resolution is generally sacrificed in the HS mode. High-resolution imaging of transient functional Ab–Ag complexes with AFM is difficult to implement.

Here, we reason that transient binding conformations of a single IgG can be observed in aqueous solution at room temperature. We devise DNA origami epitopes (DOEs) to elucidate the transient binding conformations of immunoglobulin Gs (IgGs, 150 kDa). Using AFM, HS-AFM and single-molecule FRET (smFRET), we interrogate the structure, avidity, and dynamic binding processes of IgGs at the single-molecule level.

## Results

### Design and fabrication of DOEs for IgG capture

**Fig. 1:**
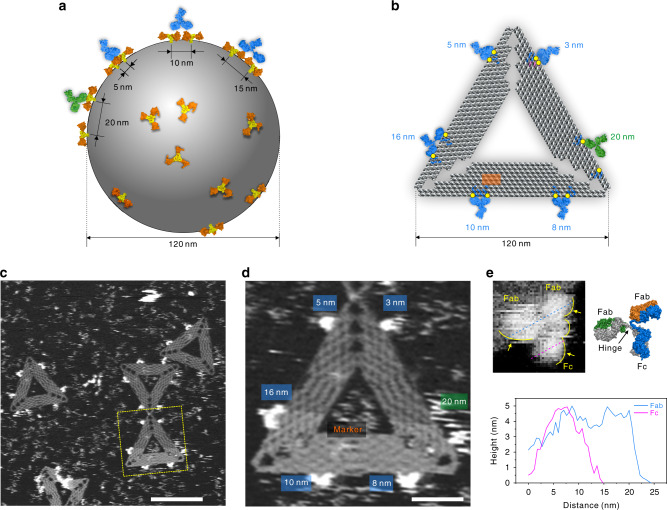
DOE-based capture of IgGs. **a** Schematic representation of non-even distribution of epitope spikes on the surface of a viral particle. **b** Designed, virus-mimicking DOEs for IgG capture and binding. Artificial epitope (digoxin, yellow), DNA origami (gray), modified staple DNA strands (blue and pink), IgGs (bivalent binding, blue; monovalent binding, green). **c** PeakForce**-**AFM image for DOE-based capture of IgGs. Scale bar, 100 nm. **d** Enlarged view of the dashed yellow square in panel **c**. Scale bar, 20 nm. **e** A high-resolution HS-AFM image of an Fc and two Fab domains of the bound IgG (upper left, yellow arrows); atomic structure of IgG (Protein Data Bank (PDB) entry 1HZH, upper right); cross-sectional profile of three domains (Fab, blue and Fc, magenta) in a DOE-bound IgG (bottom). Source data are provided as a Source Data file.

Epitope spikes often distribute unevenly on the surface of viral particles (Fig. 1a), which has been known to influence the avidity of antibodies^32,33^. We were inspired to design DNA origami^34–44^-based DOEs mimicking the distance distribution of viral epitopes to program the antibody-binding ability (Fig. 1b). To do that, six pairs of digoxin molecule (780 Da) were site specifically anchored on the prescribed positions of a triangular DNA origami. The lateral distances of each epitope pair were separated by 3–20 nm (Supplementary Figs. 1 and 2).

Having established the DOE platform, we performed IgG capture which was imaged with AFM. DOEs were deposited onto a freshly cleaved mica substrate, which was then incubated with IgGs. Figure 1c shows a solution-phase AFM image of IgG-bound DOEs. We observed Y-shaped bright dots representing IgGs with various binding structures located on all prescribed sites of DOEs. Interestingly, on sites with the epitope distances of 8, 10, and 16 nm, the three arms of IgGs were clearly visible; however, the three-arm structures of IgGs were not well defined on sites with the epitope distances of 3, 5, and 20 nm (Fig. 1d), exhibiting epitope distance-dependent binding behaviors. Especially, the image of an IgG was blurred for the lateral distance of 20 nm, suggesting that the bound IgG was mobile. In this situation, the lateral distance exceeds the contour length of the two arms, which possibly only supports monovalent binding of IgGs.

The high resolution of IgGs probably arises from the firm binding of DOEs, which restricts the motions of IgGs when they bivalently bind to the epitope pair (avidity of 2). Importantly, the stiff DOEs not only rigidify IgGs but prevent their random adsorption on mica. The near lying-flat orientation of IgGs provides a precise tool for measuring the size and conformational variations at different binding states at the single-molecule level. The Y-shaped structure and the size of the three arms of IgGs measured to be most 4.0 × 4.0 × 5.5 nm^3^ (Supplementary Fig. 3) fit well with the molecular structure of IgGs obtained from the Protein Data Bank (PDB entry, 1HZH) and the topographical AFM image of IgGs from electron tomography (ET) and AFM^11,26,45^. We assumed the two bound arms were the Fab domains where the unbound one was the Fc domain (Fig. 1e). We assigned the bound two arms as Fab regions. We also note that the hinge domain was visible, as shown in Fig. 1e.

### Conformational flexibility of DOE-confined IgGs

**Fig. 2:**
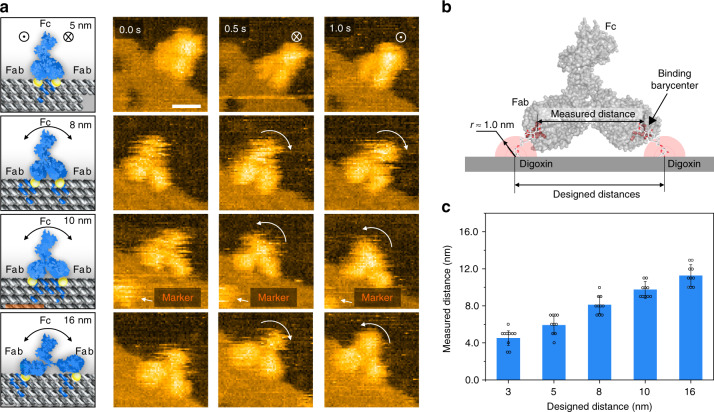
HS-AFM characterization of the conformational flexibility and measured Fab–Fab distance of DOE-confined IgGs. **a** Schematics representing the distinct conformations of single IgGs captured by DOEs with designed distances of 5, 8, 10, and 16 nm, respectively (left). Snapshot HS-AFM images (2 fps) of single IgGs bound to various designed lateral distances of epitopes (right). Different conformations of IgGs, and positional fluctuations of the mass barycenters of Fc domains, are observed (right, white arrows). Scale bar, 10 nm. **b** Schematic illustration of the measured distances between the barycenters of two Fab domains, and designed distances of paired epitopes (black lines). **c** Relationship between the designed digoxin distances (3, 5, 8, 10, and 16 nm) and measured distances of Fabs in IgGs (4.6 ± 1.0, 6.0 ± 1.0, 8.1 ± 1.0, 9.7 ± 0.8, and 11.3 ± 1.2 nm). Central values represent average values, and error bars represent the standard deviations, as calculated from independent experiments (*n* =  10). Source data are provided as a Source Data file.

Having established the DOE capture system, we next explored the conformational flexibility of DOE-confined IgGs with HS-AFM. Figure 2a shows HS-AFM snapshots (2 frame s^−1^) of IgGs bound to several DOEs sites (5, 8, 10, and 16 nm). We observed that IgGs confined in all the four separation distances showed wagging motions, as indicated by the arrows in Fig. 2a. Generally, two types of wagging motions exist: out-of-plane wagging (the 5 nm site, white circles) and in-plane wagging (8, 10, and 16 nm sites, white arrows). The former appears to be a whole-body motion when the two Fab domains are held tight, whereas the latter is primarily the motion of the Fc domain (Supplementary Figs. 4 and 5). We also note that this flexibility of binding conformations of IgGs is likely of relevant physiological functions^46–51^.

Next, we examined the relationship between the measured distance of Fab barycenters and the epitope distances (Fig. 2b). Despite the dynamic motions of IgGs, we found that the distance of Fab barycenters were nearly fixed, providing a precise measure for the stretching of Fab domains. As a general trend, the measured distances increased along with the designed epitope distances, suggesting that the two Fab domains of a single IgG could be programmably stretched using epitope distance-engineered DOEs. Of note, we found the deviation of two distances especially at the longer distances. The measured distances of two Fabs in IgGs are 4.6 ± 1.0, 6.0 ± 1.0, 8.1 ± 1.0, 9.7 ± 0.8, and 11.3 ± 1.2 nm for the designed digoxin distances 3, 5, 8, 10, and 16 nm, respectively (*n* = 10, central values = average values, and ± = standard deviations (SD)) (Fig. 2c, Supplementary Fig. 6). This is possibly because the measured distance is the distance between the barycenters of two Fabs, which is smaller than the epitope distances, especially when the two Fabs are stretched (e.g. at an epitope distance of 16 nm). Overall, we showed that DOEs could program the stretching states of IgGs, and image the stretching conformations at room temperature.

### Dynamics of DOE-IgG binding

**Fig. 3:**
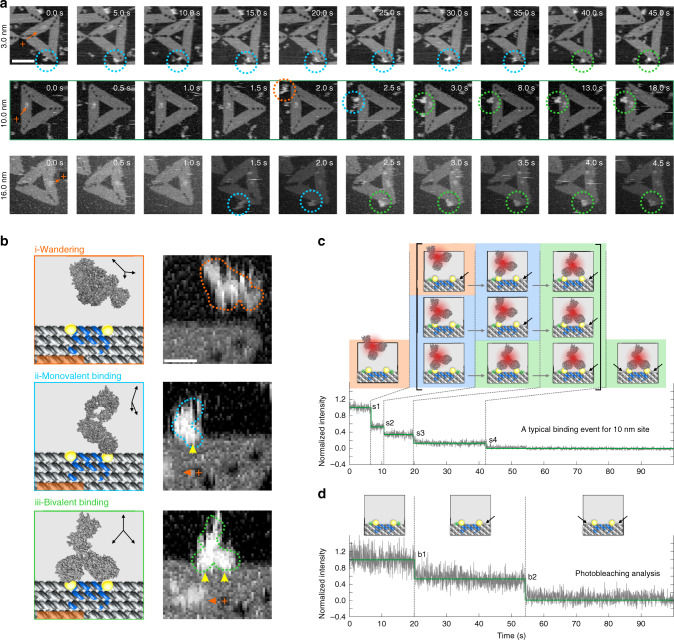
Dynamics of IgG binding to DOEs. **a** HS-AFM images for temporal evolution of the three-stage binding process, from wandering (orange circle) to monovalent binding (cyan circle), then bivalent binding (green circle) states for DOEs with designed distances of 3, 10, and 16 nm, respectively. Scale bar, 60 nm. **b** Schematic of the three-stage binding process and corresponding HS-AFM images (10-nm site: representative images of over ten independent replicates). The black arrows within each coordinate indicate the orientation of the IgG model. The dashed contours highlight the observed IgGs at different stages. The yellow arrows indicate the binding sites. The orange arrows indicate the maker, and orange plus signs represent positive orientation of DOE platform. Scale bar, 10 nm. **c** Exemplary single-molecule FRET trajectory (gray, raw 31.25 Hz data), and hidden Markov model fit (green) of a binding event for 10 nm site. A pair of ATTO 550 dye (donor, green dots) is labeled to the proximity of the epitopes. Alexa 647 dye (acceptor, crimson dots) is labeled on IgG. Note that two photobleaching events are inserted in the three-stage binding process observed by HS-AFM, resulted in a total five fluorescence intensity levels. The second bleaching could only occur at s4, while the first bleaching may occur at s1, s2, or s3. **d** Photobleaching analysis of the DOEs only system showing two-step (b1 and b2) signal drop due to the successive bleaching of two ATTO 550 dyes. Source data are provided as a Source Data file.

To probe the DOE-IgG-binding dynamics, we employed HS-AFM (2 frames s^−1^) to trace transitional binding of IgGs on tens of DOEs (Fig. 3a, Supplementary Fig. 7). We observed a three-stage DOE-IgG-binding process: (i) wandering state, (ii) monovalent binding, and (iii) bivalent binding state (Fig. 3a). Figure 3b shows three exemplary binding events for the 3, 10, and 16 nm sites on DOEs. Especially, for 10 nm site (middle raw), initially, we found the IgG molecule wandering in the bulk solution out of plane until 2.0 s (highlighted by orange circle). Then, in <0.5 s, one Fab arm of the IgG was captured by an epitope spike (highlighted by cyan circle). Finally, within another 0.5 s, the other Fab arm was captured by the proximal epitope spike. Then the two Fab arms were firmly held by the epitope pair, leaving a wagging Fc domain (highlighted by green circles). The phenomena that IgG underwent three typical conformational states before achieving bivalent binding were observed in various designed sites, except on the 20 nm site. It should be noted that 10 nm sites had the shortest monovalent-to-bivalent binding intervals of ~1.0 s (*n* = 7, SD = 0.5 s) (Supplementary Table 7, Supplementary Fig. 8). For other distances, the intervals were generally ~85.0 s (3 nm, *n* = 5, SD = 46.0 s), ~49.0 s (5 nm, *n* = 9, SD = 31.0 s), ~3.0 s (8 nm, *n* = 5, SD = 2.4 s), and ~3.0 s (16 nm, *n* = 3, SD = 4.4 s), respectively (Supplementary Table 7, Supplementary Figs. 10, 12, 14, and 16). In addition, the dynamic motions of the Fab arms are illustrated in detail with magnified HS-AFM images and movies (Supplementary Figs. 9, 11, 13, 15, and 17, Movies 1–5).

Next, we employed total internal reflection fluorescence (TIRF) microscopy to perform smFRET to validate the three-stage DOE-IgG binding mechanism. To this end, Alexa 647 acceptor was labeled on the IgG, a pair of ATTO 550 donors was located proximal to the epitope spikes (10 nm) on DOE. Figure 3c shows successive step-down fluorescence trajectory in a typical DOE-IgG-binding event. Given that photobleaching of the two ATTO 550 dyes generated only two descending steps with intervals generally longer than 10 s (Fig. 3d, Supplementary Fig. 18), the initial fluorescence intensity descending steps at ~7 s and ~10 s were assigned to a two-step FRET change brought by IgG monovalent and bivalent binding states (Fig. 3c, insertions). Generally, the smFRET analysis complements HS-AFM to establish the three-stage DOE-IgG binding mechanism (Supplementary Figs. 18 and 19).

### Engineering DOEs for evoking the IgG avidity

**Fig. 4:**
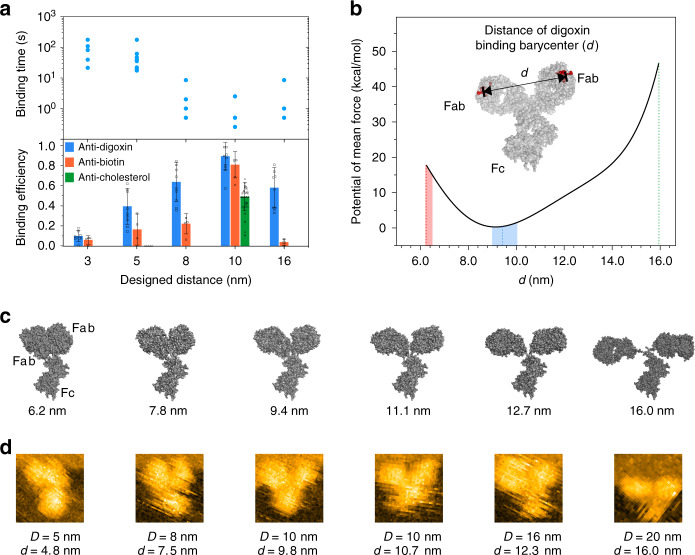
Engineering DOEs for evoking the IgG avidity. **a** Scatter plot for the monovalent-to-bivalent-binding transition kinetics of IgG-DOE (upper, HS-AFM), and histogram of IgG binding efficiency as a function of the designed distance on DOEs (bottom, PeakForce-AFM). The error bars represent the standard deviation, calculated from three independent experiments for each type of IgG. For the anti-digoxin, anti-biotin, and anti-cholesterol IgG types, the numbers of DNA origami used for each DOE were 252, 372, and 661, respectively. **b** Course-grained molecular dynamics calculations. Calculated energy diagram for IgG binding between mass barycenters at various distances. The most preferred binding site distance of IgGs was 9.5 nm. Conformations with binding site distance short or longer than 9.5 nm are all energy unfavorable. Especially, when distance longer than 15.0 nm, the potential of mean force was dramatically increased. It also implies that conformations with or close to 9.5 nm has the largest probability of occurrence. **c**, **d** Typical simulated free IgG conformations using course-grained molecular dynamics calculations and corresponding HS-AFM images for the bivalently bound IgGs on DOEs. Source data are provided as a Source Data file.

To quantify the epitope distance effect on the IgG avidity, we explored the transition binding kinetics of IgGs from monovalent (avidity of 1) to bivalent (avidity of 2) using HS-AFM. The transition time varied greatly from sub-second to over 100 second at different epitope distance. We found that the 10 nm site led to the shortest transition time (Fig. 4a, upper), implying a preferable distance for bivalent binding of IgGs. Shortened distances (3, 5, and 8 nm) resulted in longer transition time, possibly due to the increased steric effects under these circumstances. Increasing the distance to 16 nm also slightly extended the transition, suggesting that stretching the two IgG arms requires significant relaxation time which is consistent with previous studies that, structurally, the two Fab arms cannot be stretched over 20 nm^33^. The epitope distance effect on the IgG avidity was further tested using PeakForce-AFM. The difference between 8 nm and 10 nm, 10 nm and 16 nm is statistically significant (*P < 0.05, ANOVA) when judged from the monovalent/bivalent binding efficiency. Compared with monovalent/bivalent binding efficiency of 19.5%/80.6% for distance of 10 nm, monovalent/bivalent binding efficiency of 32.7%/60.1% and 47.1%/53.3% correspond to distances of 8 and 16 nm, respectively (Supplementary Fig. 20), indicating that it is more difficult to achieve bivalent binding at both 8 and 16 nm as compared to 10 nm distance.

The bivalent binding efficiency was further evaluated with different epitope distances (Fig. 4a, bottom, Supplementary Figs. 20–24). Consistent with the transition kinetics, we found that the 10 nm distance led to the highest bivalent binding efficiency, whereas shorter or longer distances decreased the efficiency. The generality of this distance-dependent-binding efficiency was substantiated with the use of biotin and anti-biotin IgG, as well as cholesterol and anti-cholesterol IgG pairs on designed DOEs (Supplementary Figs. 25–32). We should note that although DNA origami enables the modification of molecules with nanometer precision, entities modified on the ends of the DNA staple strand may endure uncertainty. For example, Shaw et al.^41^ showed that the optimal distance for IgG binding to two epitopes is 16 nm, whereas our results showed that this distance is about 10 nm. Different uncertainty estimation method, characterization technique, and design strategies for DNA origami that cause extensions at binding sites are presumable reasons for explaining this discrepancy (Supplementary Fig. 2).

To understand the mechanism of the epitope distance-dependent avidity of IgGs, we performed coarse-grained molecular dynamics (MD) simulations on IgG-DOE binding. Interestingly, we found that IgG had the lowest potential of mean force (PMF) at a binding barycenter distance *(d)* of ~9.4 nm (Fig. 4b). According to the PMF equation:

1 $${\mathrm{{PMF}}} = -kT{\mathrm{{ln}}}P(r),$$

where *P(r)* is the probability that a certain IgG conformation forms at a given *d, k* is the Boltzmann constant, and *T* is the system temperature. Hence, conformations at ~9.4 nm have the highest probability of occurrence and the lowest potential, which is consistent with the experimental results that the 10 nm sites are kinetically and thermodynamically favored. The PFM profile shows that conformations with *d* smaller or greater than 9.4 nm were all energetically unfavored. In other words, the favored ~9.4 nm conformation at equilibrium corresponds to the average value of the Fab–Fab angle ~ 105°. This finding is in good agreement with previous structure analysis of the intact IgGs with Fab–Fab angle ~ 110° in cryo-EM^46^. Note that when *d* > 16 nm, the molecular structure of IgG start to collapse during computation. Taking the active radius into consideration, the maximum stretching length of IgGs is no more than ~18 nm, which is consistent with our experimental observations.

We further employed MD simulations to compute the dynamic conformations of bivalently bound IgGs (Fig. 4c). We found that IgG could take conformations ranging from highly compact (*d* = 6.2 nm) to far stretched (*d* = 16.0 nm). The design of epitope distances on DOEs provides a straightforward and programmable approach to observe these transient, functional conformations at room temperature. Of note, the measured barycenter distance *d* is smaller than the designed distance D. Importantly, we imaged these transient conformations of IgGs in solution with AFM, which were all captured at prescribed distances (Fig. 4d).

## Discussion

In this study, we devised DOEs to mimic the distance distribution of epitopes on viral particles by exploiting the spatial addressability of DNA origami. The positioning ability and stiffness of DOEs enables room-temperature freezing of IgGs for high-resolution imaging of transient, functional IgG binding conformations at the single-molecule level. The precision in programmable control of the lateral distances of epitopes on DOEs allows unequivocal determination of the epitope distance-dependent IgG avidity. This DOE platform also supports HS-AFM and smFRET analysis to probe the dynamics of single IgG binding events on DOEs.

The dynamics of Ab–Ag binding has been extensively studied theoretically and experimentally^24,52–56^. However, direct capture of transient binding conformations of Ab–Ag complexes at room temperature remains difficult. Our DOE platform provides direct structural evidence for the transient, functional conformations of IgGs at room temperature, which may deepen our understanding of physiologically or pathologically relevant Ab–Ag complexes. Especially, we find that IgGs can take flexible conformations ranging from high compact to far stretched in response to short-to-long epitope distances. Importantly, the binding kinetics and efficiency for bivalent IgG binding is the highest when the two epitopes are separated by ~10 nm.

The distance-dependent binding of IgGs on epitopes has important physiologically or pathologically relevance. Viruses have been well known to adopt wise strategies to escape Ab-mediated neutralization strategy by tuning the spatial distribution of epitope spike on their surfaces^32^, e.g. average spike distances on the surface of HIV is >20 nm, which is far beyond the span of two Fabs in a single Ab molecule^33^. Consequently, the flexibility of IgG binding with the two arms is critically important for their affinity/avidity^51^. The designability and programmability of DOEs thus offer an intuitive method to imitate viral epitope distribution. DOEs thus not only increase the design space for understanding Ab–Ab interactions at the single-molecule level but also provide a potentially powerful platform for engineering immunological tools.

## Methods

### Materials

The long single-stranded M13mp18 DNA molecule was obtained from New England Biolabs (NEB). Digoxin-labeled, biotin-labeled, cholesterol-labeled, cholesterol-modified poly A (chol-A), and the rest of staple strands were bought from Sangon Biotech Co., Ltd (Shanghai, China). ATTO 550-labeled DNA short staple strands were bought from Takara, China. Anti-digoxin antibody and anti-biotin antibody were obtained from Sigma Aldrich, China. Anti-cholesterol antibody was brought from Lifespan Bioscience, Inc. 1,2-Dioleoyl-sn-glycero-3-phosphocholine (DOPC) and 1,2-dimyristoyl-sn-glycero-3-phosphocholine (DMPC) were obtained from Avanti Polar Lipids, USA. Alexa Fluor 647-IgG (Alexa 647-IgG) was obtained from the Jackson laboratory, USA.

All reagents were kept at −20 °C until use. Deionized (DI) Water used in the experiments was 18.2 MΩ cm^−1^. Milli-Q water was generated by a Millipore system. Polycarbonate (PC) membrane (Whatman, Fisher Scientific) with a pore diameter of 100 nm was used in vesicle extrusion. Millipore was obtained from GE Healthcare (Little Chalfont, UK). The PCR instrument used was the Eppendorf Mastercycler Personal Machine. The concentration of DNA origami was measured with NanoPhotomer-P330 (IMPLEN, Munich, Germany).

### Design and fabrication of DOEs for IgG capture

The DOE comprised a triangular DNA origami tile fixed with epitopes. The sites and sequences of the staple strands for generating the DNA origami have been previously reported^34^. The DOEs were based on paired distances of digoxins, biotins, or cholesterols on the DNA origami. To obtain potentially accurate distance for the paired epitopes, all epitopes were modified directly at the 5′-, 3′- and/or interval of the staple strand on the DNA origami, and no extra linker was added. The designed distances of the paired epitopes (approximately 3, 5, 8, 10, 16, and 20 nm) were based on the numbers of nucleotides within the DNA origami: a one-turn helix (32 nt) had a length of 10.6 nm. However, some uncertainties can be expected due to the flexibility of the helix structure, the size of epitope, and considering that the B-helix structure has a distance of 0.34 nm per base pair and a 2.0 nm diameter per helix, we estimated that the spatial variance of the designed distance was <3.0 nm. Therefore, all lateral distances are described in terms of their design distances, unless mentioned.

The sites and sequences of (1) the staple strands labeled with digoxin, biotin, cholesterol, and ATTO 550; (2) the extended sequences of staple strands for anchoring DNA origami on a supported lipid bilayer (SLB); and (3) the DNA origami makers used are listed as Supplementary Tables 1–6, respectively. All staple sequences listed in the tables are presented in 5′–3′ order.

### Constructing a DOE based on assembly with digoxin, or biotin, or cholesterol using DNA origamis

The DOE was constructed by assembly of triangular DNA origami tile, with digoxin/biotin/cholesterol-labeled replacing strands. For smFRET experiments, strands labeled with ATTO 550 and extended by 20-dT were added during the self-assembly process. Self-assembly was conducted in TAE/Mg^2+^ buffer (40.0 mM Tris, 2.0 mM EDTA, and 12.5 mM MgCl_2_, pH 8.0)^57^. Briefly, a 5.0 nM single-stranded M13mp18 DNA, a 10-fold molar excess of staple strands, and 10-fold molar excess of digoxin, biotin, or cholesterol-labeled staple strands were mixed, followed by annealing treatment from 95 °C to 4 °C at a speed of 0.1 °C per 10 s. The excess staple strands were removed by ultrafiltration three times using 1× TAE/Mg^2+^ buffer using 100 kDa cutoff filters (Amicon).

For preparing the DOEs used in the single-molecule smFRET experiments, single-stranded M13mp18, a 10-fold molar excess of staple strands, a 10-fold molar excess of digoxin, and a 400-fold molar excess of ATTO 550-labeled strands were mixed, followed by the same annealing and ultrafiltration process described above.

### Preparation of the SLB

The small unilamellar vesicles composed of DOPC or DMPC were generated^58^. A particular volume of chloroform solution of DOPC or DMPC was placed in a 25 ml round-bottomed flask. Then, the lipid film was dried under a stream of N_2_ using a rotary evaporator to evaporate the chloroform. The thoroughly dried mixture was immersed in DI water and sonicated for 10 min. A lipid solution (5 mg ml^−1^) was extruded through a pore of polycarbonate membrane (100 nm in diameter) >30 times. The resulting small unilamellar vesicles solution was used immediately.

An SLB was generated on a cover glass by fusion of small unilamellar vesicles. The cover glass used to support the SLB glass was dried using N_2_ after sonicating for 15 min in chloroform, acetone, ethanol, KOH, and DI water successively to avoid influence of fluorescence background. Then, a quadrate chip fence in an edge length of 0.5 cm was attached to the cover glass to construct a sample chamber, using 50 µl of small unilamellar vesicles solution for incubation for 30 min at 25 °C. Excess unfused small unilamellar vesicles were removed by thoroughly rinsing with DI water.

### Tethering of the DOE on the SLB

Poly-A staple strands (400 nM) were added to the prepared SLB for 1 h incubation at 25 °C. Then, the DOE solution (100 pM) was added for 30 min incubation at 25 °C, after washed by 1× TAE to remove the excess poly-A staple strands. The resulting tethered DOEs on the SLB were ready to use after the excess DOEs were washed away using 1× TAE buffer.

### AFM sample preparation

To quantify binding efficiency, experiments of DOE-bound IgGs were conducted in solutions, in which the concentration of the DNA origami in the reaction was kept in below 0.2 nM. Anti-digoxin antibody (Catalog Number: D8156) was diluted 800 to 3200 times with TAE buffer before reaction with an equal volume DOE solution. Anti-biotin antibody (Catalog Number B7653) and anti-cholesterol antibody (Catalog Number LS-C295824) were diluted with TAE buffer into final concentration from 30 to 90 nM and 78 to 312 nM, respectively.

### HS-AFM imaging

HS-AFM Images were collected using tapping mode HS-AFM (RIBM, Japan). Silicon cantilevers (AC40, Olympus, Tokyo, Japan) with nominal spring constants of 0.09 N m^−1^ and a resonance frequency of 110 kHz were used. HS-AFM was performed at room temperate (~25 °C).

To capture the dynamic epitope-IgG binding process, 5 µl of digoxin-labeled DNA origami was incubated on freshly cleaved mica for 5 min. The sample was mounted on the AFM liquid cell filled with 30 µl 1× TAE-Mg^2+^ imaging buffer. After capturing an intact DNA origami, a final diluted ratio 1:10,000 IgG in 1× TAE buffer was added to the liquid cell by pumping. Images were captured at a rate of 0.5 s with a scan area of 150 × 150 pixel^2^. To obtain high-resolution images of IgG conformation, images were captured at a rate of 0.1 s at a scan area of 70 × 70 pixel^2^.

### HS-AFM data analysis

The HS-AFM data were analyzed using kodec analysis software. The HS-AFM movies were drift corrected and contrast adjusted.

### PeakForce-AFM imaging

Images were collected in PeakForce tapping mode (AFM MultimodeVIII, Veeco, Plainview, NY, USA). Cantilevers with nominal spring constants of 0.12 N m^−1^ (Sharp Silicon Nitride Lever (PEAKFORCE-HIRS-F-B; Bruker, Billerica, MA, USA)) were applied using a typical scanning speed of 1–2 Hz. To capture images of the three types of Ab–Ag complexes formed in solution, DOEs were mixed with IgGs in 1× TAE buffer (pH 8.0). After 2 h incubation at 25 °C, a 3 µl drop of the formed Ab–Ag complex solution was adsorbed on a freshly cleaved mica surface for incubation for 5 min. The resulting samples were mounted in liquid cell in 1× TAE/Mg^2+^ buffer for imaging. All images were flattened and analyzed using NanoScope Analysis software.

### smFRET measurement

All smFRET experiments were performed using an ATTO 550-Alexa 647 coupling system in which each DNA origami was modified with one ATTO 550 molecule near one digoxin molecule using a distance of 10 nm.

### smFRET on lipid bilayer

All smFRET experiments were performed on a commercial TIRF microscope (N-storm, Nikon) using a ×100 objective lens (NA 1.49) and an electron multiplying charge-coupled devices (EMCCD) camera (iXon 3, Andor). A solid-state laser operating at 561 nm (200 mW) and 640 nm (200 mW) was used to excite the fluorescence of ATTO 550 and Alexa 647, respectively. ATTO and Alexa signals were collected separately.

The reaction fluorescence signals were obtained as following: Firstly, we localized an imaging region (40 × 40 μm^2^) in which several static bright spots were observed using 561 nm laser excitation. Then, Alexa-labeled IgGs were immediately added to the sample chamber, and the fluorescence intensity of ATTO 550 was recorded with a 40 ms time resolution. The obtained videos were used as for subsequent signal of smFRET.

### smFRET data analysis

Image analysis was carried out using Image J software. The Bio-Formats plugin was used to extract co-localized ATTO and Alexa spots to obtain the FRET signal. Individual ATTO–Alexa pairs that dropped in successive steps revealed changes in fluorescence intensity during IgG binding. These pairs are described in Supplementary Figs. 18 and 19, which also provide the distribution of fluorescence intensity of the ATTO 550-DNA-origami after Alexa-IgG binding.

Hidden Markov Model (HMM) is widely used for identifying hidden states from noisy traces, especially in single-molecule FRET analysis. Which is specified by the following components: a set of *N* states (*Q* = *q*_1_ *q*_2_ … *q*_*N*_), a transition probability matrix *A* (*A* = *a*_11_ … *a*_*ij*_ … *a*_*NN*_, each *a*_*ij*_ representing the probability of moving from state *i* to state *j*), a sequence of observations *O*, a sequence of emission probabilities *B*, an initial probability distribution over states *π*. State assignment was performed using open source hmmlearn package (https://pypi.org/project/hmmlearn/) in Python 3.6. Observed fluorescence intensity sequence was read in as *O*. *Q* was initiated with a sequence of intensity values with equal intervals from minimum to maximum of observed intensity. Transition matrix *A* was randomly generated initially. Initial probability distribution was assumed as a uniform distribution on all states.

### Coarse-grain MD simulation

*Initial conformation generation*: The CG model of IgGs was converted from full-atom structure (pdb number: 1igt) with parameters of martini CG version 2.2 (refs. ^59,60^) and gromacs_2016 (ref. ^61^). Then, the model was put into a 40 nm^3^ water box. After minimization and equation for 10 ns at 300 K using NPT ensemble, we pushed two Fabs together by applying 1 kJ mol^−1^ force on the binding site of digoxin at a rate of 0.1 nm per 1000 ps. The distance of the binding site was varied from 14 to 5 nm. Then, 10 ns equation simulation was carried out to remove unrealistic conflict. The final distance between binding sites of two Fabs was 5.4 nm. The binding site is constituted by residues: A_Ser34, A_Gln89, A_Gly91, A_Tyr94, B_Tyr35, B_His95, B_Gly97, B_Tyr100, and corresponding residues in chain C, D. The distances between two binding sites was counted by measuring the mass centers of the two groups. In all simulation, a position restraint of 1000 kJ mol^−^^1^ was applied on backbone beam of Fc part. To hold the conformation stability of Fabs, elastic network (between beams with distance in the range of 0.5–2 nm) with a force of 500 kJ mol^−1^ was applied. Moreover, to insure the flexibility of linker parts (residue B230–B250, D230–D250) between Fc and Fabs, no restraint was implemented in linker parts.

### Umbrella sampling

Umbrella sampling is a method that calculates free energy along a reaction coordinate based on MD simulation sampling. We used weighted histogram analysis method (WHAM) to estimate the PMF of distance changing of Fabs. Pulling was initialed with the conformations that have a distance of 5.4 nm from two binding sites. The pulling force was set to 1 kJ mol^−^^1^ and pulling rate was set to 0.01 nm per 100 ps. The distance was altered from 5.4 to 17.3 nm. Snapshot of every 0.1 nm was fetched to perform 1 ns harmonic constraint MD simulation. A harmonic potential, *f(x)* = *−kx*^2^ (*k* = 100 kJ mol^−1^ nm^−2^), was applied. The 12.0 nm distance was evenly divided into 120 windows. The conformation sampled of each window was used for PMF calculations with WHAM. The result was presented in Fig. 4b.

### Reporting summary

Further information on research design is available in the Nature Research Reporting Summary linked to this article.

## Supplementary information

Supplementary information

Description of Additional Supplementary Files

Supplementary Movie 1

Supplementary Movie 2

Supplementary Movie 3

Supplementary Movie 4

Supplementary Movie 5

Reporting Summary

## Data Availability

The authors declare that the main data supporting the findings of this study are available within the article and Supplementary Information files. Extra data are available from the corresponding author upon request. The source data underlying Figs. 1e, 2c, 3c, d, and 4a, b and Supplementary Figs. 1d, 3b, 6d, 18b,d, 19a–c, 20a, b, 24b, 28b, and 32b are provided as a Source Data file. Source data are provided with this paper.

## Source data

Source Data
