# Supplementary material for: Capturing transient antibody conformations with DNA origami epitopes
Source: Nat Commun. 2020 Jun 19;11:3114. doi: 10.1038/s41467-020-16949-4 (PMC7305102; doi:10.1038/s41467-020-16949-4)
Supplement: Supplementary file 1 — Supplementary information [file 41467_2020_16949_MOESM1_ESM.pdf]

Supplementary Information

Capturing transient antibody conformations with DNA origami epitopes

Zhang et al.

Item	Description	Page
**Suppl. Table 1**	The sites and sequences of replaced staple strands labeled with digoxin	**4**
**Suppl. Table 2**	The sites and sequences of replaced staple strands labeled with biotin	**5**
**Suppl. Table 3**	The sites and sequences of replaced staple strands labeled with cholesterol	**6**
**Suppl. Table 4**	The sites and sequences of replaced staple strands labeled with ATTO 550 molecules	**7**
**Suppl. Table 5**	The sequences of replaced staple strands for anchoring DNA origami on the SLB	**8**
**Suppl. Table 6**	The sequences of DNA strands for the origami marker	**10**
**Suppl. Table 7**	Binding time of antibody to antigen	**11**
**Suppl. Figure 1**	Design diagram and PeakForce-AFM image of DOEs	**12**
**Suppl. Figure 2**	Schematic diagram of different ways to modify antigens	**13**
**Suppl. Figure 3**	Measurements of the three IgG domains	**14**
**Suppl. Figure 4**	Typical HS-AFM images of IgGs with different wagging motions	**15**
**Suppl. Figure 5**	HS-AFM images of Fc wagging motion in twenty two independent experiments	**16**
**Suppl. Figure 6**	Measured distances of the two binding Fabs within a single IgG molecule	**17**
**Suppl. Figure 7**	HS-AFM images recording the intermediate conformations of IgGs	**18**
**Suppl. Figure 8**	Dynamic process of digoxin-IgG complex formation in a lateral distance of 10 nm	**20**
**Suppl. Figure 9**	Enlarged HS-AFM images of dynamic conformations and orientations of IgGs for the lateral distance of 10 nm	**21**
**Suppl. Figure 10**	Dynamic process of digoxin-IgG complex formation in a lateral distance of 3 nm	**22**
**Suppl. Figure 11**	Enlarged HS-AFM images of dynamic conformations and orientations of IgGs for the lateral distance of 3 nm	**23**
**Suppl. Figure 12**	Dynamic process of digoxin-IgG complex formation in a lateral distance of 5 nm	**24**

**Suppl. Figure 13**	Enlarged HS-AFM images of dynamic conformations and orientations of IgGs for the lateral distance of 5 nm	**25**
**Suppl. Figure 14**	Dynamic process of digoxin-IgG complex formation in a lateral distance of 8 nm	**26**
**Suppl. Figure 15**	Enlarged HS-AFM images of dynamic conformations and orientations of IgGs for the lateral distance of 8 nm	**27**
**Suppl. Figure 16**	Dynamic process of digoxin-IgG complex formation in a lateral distance of 16 nm	**28**
**Suppl. Figure 17**	Enlarged HS-AFM images of dynamic conformations and orientations of IgGs for the lateral distance of 16 nm	**29**
**Suppl. Figure 18**	smFRET signals recording the binding processes of IgGs to DOEs	**30**
**Suppl. Figure 19**	Analysis of FRET data	**31**
**Suppl. Figure 20**	Effect of epitope distance on IgG avidity.	**32**
**Suppl. Figure 21**	PeakForce-AFM images of digoxin-IgG complexes in a IgG dilution ratio of 1:6400	**33**
**Suppl. Figure 22**	PeakForce-AFM images of digoxin-IgG complexes in a IgG dilution ratio of 1:3200	**34**
**Suppl. Figure 23**	PeakForce-AFM images of digoxin-IgG complexes in a IgG dilution ratio of 1:1600	**35**
**Suppl. Figure 24**	Binding efficiency of digoxin-IgG complexes with various concentrations of IgGs	**36**
**Suppl. Figure 25**	PeakForce-AFM images of biotin-IgG complexes in a IgG concentration of 30 nM	**37**
**Suppl. Figure 26**	PeakForce-AFM images of biotin-IgG complexes in a IgG concentration of 60 nM	**38**
**Suppl. Figure 27**	PeakForce-AFM images of biotin-IgG complexes in a IgG concentration of 90 nM	**39**
**Suppl. Figure 28**	Binding efficiency of biotin-IgG complexes with various concentrations of IgGs	**40**
**Suppl. Figure 29**	PeakForce-AFM images of Cholesterol-IgG complexes in a IgG concentration of 78 nM	**41**
**Suppl. Figure 30**	PeakForce-AFM images of Cholesterol-IgG complexes in a IgG concentration of 156 nM	**42**
**Suppl. Figure 31**	PeakForce-AFM images of Cholesterol-IgG complexes in a IgG concentration of 312 nM	**43**
**Suppl. Figure 32**	Binding efficiency of cholesterol-IgG complexes with various concentrations of IgGs	**44**
**Suppl. Movie 1**	Dynamics of IgG binding to lateral digoxin distance 3 nm	

Suppl. Movie 2 Dynamics of IgG binding to lateral digoxin distance 5 nm

Suppl. Movie 3 Dynamics of IgG binding to lateral digoxin distance 8 nm

Suppl. Movie 4 Dynamics of IgG binding to lateral digoxin distance 10 nm

Suppl. Movie 5 Dynamics of IgG binding to lateral digoxin distance 16 nm

Supplementary Tables

Supplementary Table 1: The sites and sequences of replaced staple strands labeled with digoxin for a group of DOEs in distances of approximately 3 nm, 5 nm, 8 nm, 10 nm, 16 nm, and 20 nm, respectively.

3 nm	C59-mix-dig	CAAGTTT**T-dig-**TTGGGGTCGAAATCGGCAAAATCCGGGAAACC
	C61-3-dig	TTCCAGTCCTTATAAATCAAAAGAGAACCATCACCCAAAT-**dig**
5 nm	B28-mix-5-dig	**dig**-GATAAAAACCAAAAT**T-dig-**ATTAAACAGTTCAGAAATTAGAGCT
8 nm	A56-mix-dig	ACAAGAAAGCAAGCAAATCAGATAACAGCCATA**T-dig-**TTATTTA
	A61-3-dig	GCGCCTGTTATTCTAAGAACGCGATTCCAGAGCCTAATTT-**dig**
10 nm	A16-5-dig	**dig**-GTCAGAGGGTAATTGATGGCAACATATAAAAGCGATTGAG
	A20-3-dig	TTGACGGAAATACATACATAAAGGGCGCTAATATCAGAGA-**dig**
16 nm	B53-mix-dig	ACCAGTCAGGACGTT**T-dig-**GGAACGGTGTACAGACCGAAACAAA
	B61-3-dig	AAAACACTTAATCTTGACAAGAACTTAATCATTGTGAATT-**dig**
20 nm	C23-5-dig	**dig**-TTAAAGGGATTTTAGATACCGCCAGCCATTGCGGCACAGA
	C30-3-dig	TAAAACATTAGAAGAACTCAAACTTTTTATAATCAGTGAG-**dig**

Supplementary Table 2: The sites and sequences of replaced staple strands labeled with biotin for a group of DOEs in distances of approximately 3 nm, 5 nm, 8 nm, 10 nm, 16 nm, and 20 nm, respectively.

3 nm	C59-mix-biotin	CAAGTTT**T-biotin**-TTGGGGTCGAAATCGGCAAAATCCGGGAAACC
	C61-3-biotin	TTCCAGTCCTTATAAATCAAAAGAGAACCATCACCCAAAT-**biotin**
5 nm	B28-mix-5-biotin	**biotin**-GATAAAAACCAAAAT**T-biotin**-ATTAAACAGTTCAGAAATTAGAGCT
8 nm	A56-mix-biotin	ACAAGAAAGCAAGCAAATCAGATAACAGCCATA**T-biotin**-TTATTTA
	A61-3-biotin	GCGCCTGTTATTCTAAGAACGCGATTCCAGAGCCTAATTT-**biotin**
10 nm	A16-5-biotin	**biotin**-GTCAGAGGGTAATTGATGGCAACATATAAAAGCGATTGAG
	A20-3-biotin	TTGACGGAAATACATACATAAAGGGCGCTAATATCAGAGA-**biotin**
16 nm	B53-mix-biotin	ACCAGTCAGGACGTT**T-biotin**-GGAACGGTGTACAGACCGAAACAAA
	B61-3-biotin	AAAACACTTAATCTTGACAAGAACTTAATCATTGTGAATT-**biotin**
20 nm	C23-5-biotin	**biotin**-TTAAAGGGATTTTAGATACCGCCAGCCATTGCGGCACAGA
	C30-3-biotin	TAAAACATTAGAAGAACTCAAACTTTTTATAATCAGTGAG-**biotin**

Supplementary Table 3: The sites and sequences of replaced staple strands labeled with cholesterol for a group of DOEs in distances of approximately 5 nm, 10 nm, and 20 nm, respectively.

5 nm	B26-3-cholesterol	CGGATGGCACGAGAATGACCATAATCGTTTACCAGACGAC-**cholesterol**
	B28-3-cholesterol	GATAAAAACCAAAATATTAAACAGTTCAGAAATTAGAGCT-**cholesterol**
10 nm	A12-3-cholesterol	CATTCAACAAACGCAAAGACACCAGAACACCCTGAACAAA-**cholesterol**
	A20-3-cholesterol	TTGACGGAAATACATACATAAAGGGCGCTAATATCAGAGA-**cholesterol**
20 nm	C20-3-cholesterol	GAATACGTAACAGGAAAAACGCTCCTAAACAGGAGGCCGA-**cholesterol**
	C30-3-cholesterol	TAAAACATTAGAAGAACTCAAACTTTTTATAATCAGTGAG-**cholesterol**

Supplementary Table 4: The sequences of replaced staple strands labeled with ATTO 550 molecules.

A12-Half-3-ATTO 550	CATTCAACAAACGCAAAGACACCAGAACACCC-**ATTO 550**
A23-Half-5-ATTO 550	**ATTO 550**-ACAAGAATGTTAGCAAACGTAGAAAATTATTC

Supplementary Table 5: The sequences of replaced staple strands for anchoring DNA origami on the SLB. The bold parts are extended sequences used for immobilizing triangle DNA origami. Last is the sequence of cholesterol-poly A for hybridization with sequence of poly T of the DNA origami.

A29-3-Poly T	CCATTAGCAAGGCCGGGGGAATTA**TTTTTTTTTTTTTTTTTTTTT**
A30-3-Poly T	GAGCCAGCGAATACCCAAAAGAACATGAAATAGCAATAGC**TTTTTTTTTTT TTTTTTTTTT**
A33-3-Poly T	CCTTTTTTCATTTAACAATTTCATAGGATTAG**TTTTTTTTTTTTTTTTTTTTT**
A39-3-Poly T	TTATCAAACCGGCTTAGGTTGGGTAAGCCTGT**TTTTTTTTTTTTTTTTTTT T**
A41-3-Poly T	TTTCCTTAGCACTCATCGAGAACAATAGCAGCCTTTACAG**TTTTTTTTTTTT TTTTTTTTT**
A46-3-Poly T	GAGCAAAAGAAGATGAGTGAATAACCTTGCTTATAGCTTA**TTTTTTTTTTT TTTTTTTTTT**
A48-3-Poly T	CACCGGAATCGCCATATTTAACAAAATTTACG**TTTTTTTTTTTTTTTTTTTT**
A58-3-Poly T	AATAGATAGAGCCAGTAATAAGAGATTTAATG**TTTTTTTTTTTTTTTTTTTT**
B11-3-Poly T	CATCCAATAAATGGTCAATAACCTCGGAAGCA**TTTTTTTTTTTTTTTTTTT TT**
B17-3-Poly T	GCAAATATTTAAATTGAGATCTACAAAGGCTACTGATAAA**TTTTTTTTTTTT TTTTTTTTT**
B25-3-Poly T	TAAAGCTATATAACAGTTGATTCCCATTTTTG**TTTTTTTTTTTTTTTTTTTT T**
B34-3-Poly T	CAACAGTTTATGGGATTTTGCTAATCAAAAGG**TTTTTTTTTTTTTTTTTTT TT**
B37-3-Poly T	ACAGGTAGAAAGATTCATCAGTTGAGATTTAG**TTTTTTTTTTTTTTTTTTT TT**
B40-3-Poly T	ATTCGGTCTGCGGGATCGTCACCCGAAATCCG**TTTTTTTTTTTTTTTTTTT TT**
B46-3-Poly T	AGGTTTAGTACCGCCATGAGTTTCGTCACCAGGATCTAAA**TTTTTTTTTTT TTTTTTTTTT**
B58-3-Poly T	ACCCCCAGACTTTTTCATGAGGAACTTGCTTT**TTTTTTTTTTTTTTTTTTT TT**
C10-3-Poly T	TAATCCTGATTATCATTTTGCGGAGAGGAAGG**TTTTTTTTTTTTTTTTTTTT T**
C17-3-Poly T	GCGCAGAGGCGAATTAATTATTTGCACGTAAATTCTGAAT**TTTTTTTTTTTT TTTTTTTT**
C25-3-Poly T	TTGAGGATGGTCAGTATTAACACCTTGAATGG**TTTTTTTTTTTTTTTTTTT T**
C34-3-Poly T	AGGAAGATGGGGACGACGACAGTAATCATATT**TTTTTTTTTTTTTTTTTTT TT**

C40-3-Poly T	GCCAGTGCGATCCCCGGGTACCGAGTTTTTCT**TTTTTTTTTTTTTTTTTTT TT**
C41-3-Poly T	TTTCACCAGCCTGGCCCTGAGAGAAAGCCGGCGAACGTGG**TTTTTTTTTT TTTTTTTTTTT**
C46-3-Poly T	GTTAAAATTCGCATTAATGTGAGCGAGTAACACACGTTGG**TTTTTTTTTTT TTTTTTTTTT**
C58-3-Poly T	TGTCGTGCACACAACATACGAGCCACGCCAGC**TTTTTTTTTTTTTTTTTTT TT**
Poly A-chol	**AAAAAAAAAAAAAAAAAAAA-cholesterol**

Supplementary Table 6: The sequences of replaced staple strands for the origami marker.

IndexA02	AGCGTCATGTCTCTGATCCTCTTTTGAGGAACAAGTTTTCTTGTATTTACCGACTACCTT
IndexA03	TTCATAATCCCCTTATTCCTCTTTTGAGGAACAAGTTTTCTTGTTAGCGTTTTTCTTACC
IndexA06	CCGGAACCCAGAATGGTCCTCTTTTGAGGAACAAGTTTTCTTGTAAAGCGCAACATGGCT
IndexA07	AAAGACAACATTTTCGTCCTCTTTTGAGGAACAAGTTTTCTTGTGTCATAGCCAAAATCA
IndexA10	TGTACTGGAAATCCTCTCCTCTTTTGAGGAACAAGTTTTCTTGTATTAAAGCAGAGCCAC
IndexA11	CACCGGAAAGCGCGTTTCCTCTTTTGAGGAACAAGTTTTCTTGTTTCATCGGAAGGGCGA

Supplementary Table 7: Binding time of antibody to antigen.

**Distance (nm)**	**Time (s)**									**Mean**	**SD**
3	81.5	21.5	39.5	109.0	177.5	---	---	---	---	85.8	46.0
5	21.0	20.5	22.0	35.5	39.5	47.0	61.5	17.5	177.0	49.0	31.0
8	0.5	2.0	1.0	0.5	8.5	---	---	---	---	2.5	2.4
10	0.5	0.5	0.5	0.5	2.5	0.5	0.25*	---	---	0.8	0.5
16	8.5	1.0	0.5	---	---	---	---	---	---	3.3	4.4

*Note that here 0.25 s means the binding time is between 0.0 s and 0.5 s, and cannot be precisely determined by HS-AFM due to the scanning speed limit.

Supplementary Figures

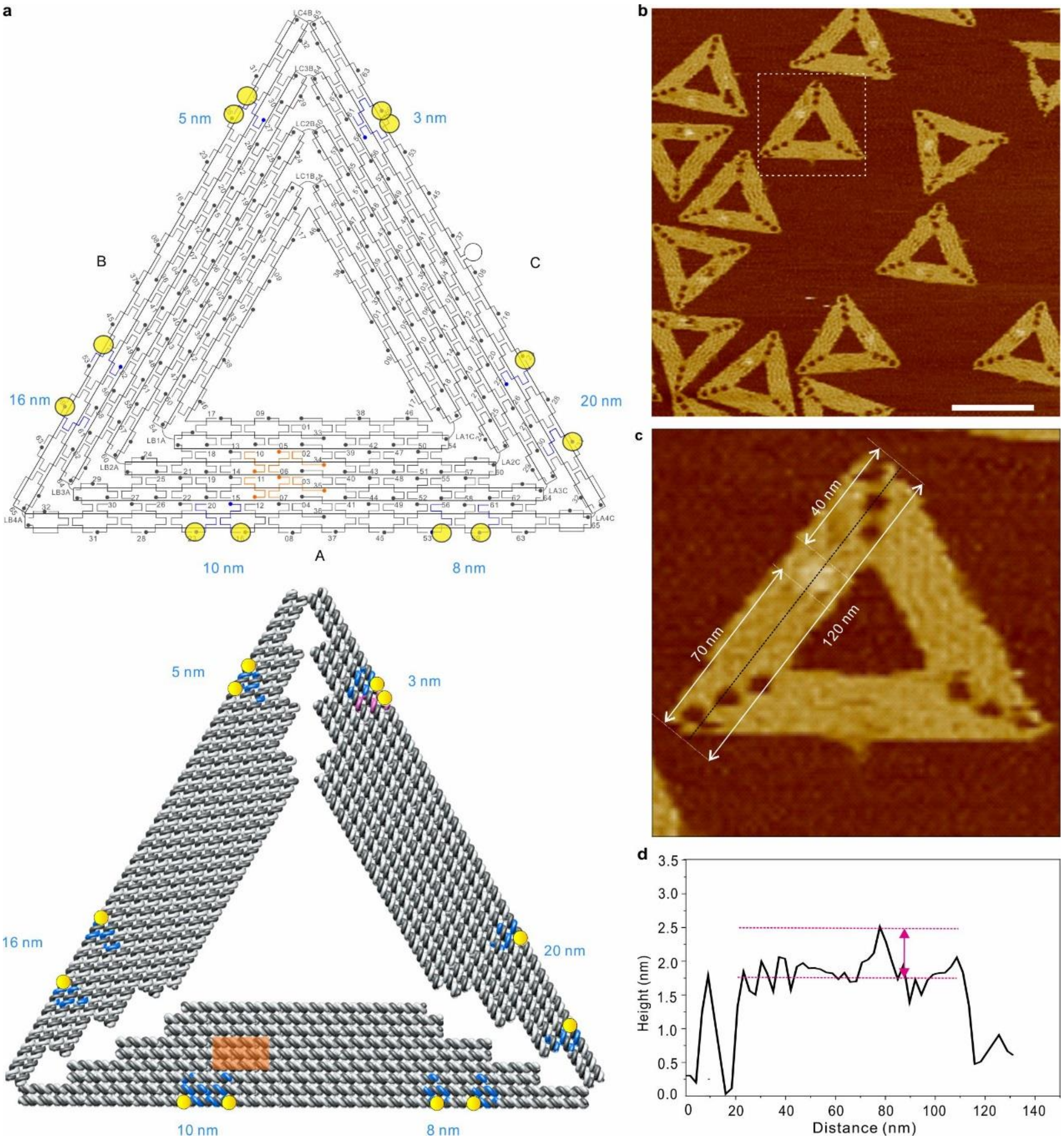

Supplementary Figure 1. Design diagram and PeakForce-AFM image of DOEs. a, Detailed design and Maya 3D rendering of DOE, which is consisted by triangular DNA origami with positioning modified digoxin. **b,** AFM image of DOEs. Scale bar, 100 nm. **c,** Enlarged view of the dotted white square in (b), where one side of the DNA origami is marked by a spot. **d,** Cross-sectional profile of the marker across the dashed black line in (c). Source data are provided as a Source Data file.

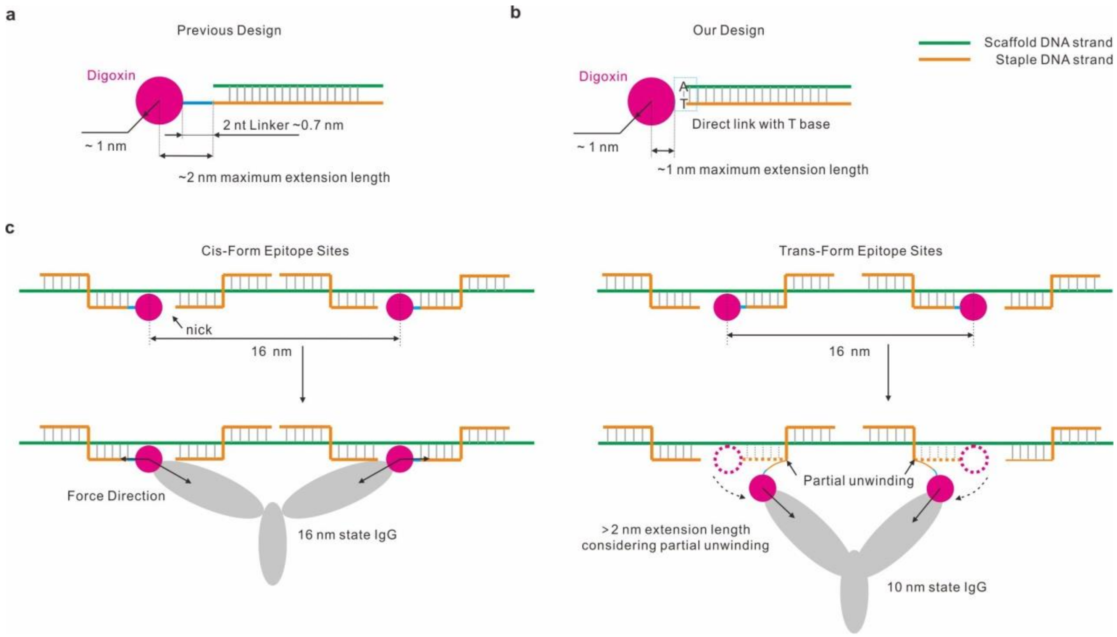

Supplementary Figure 2 Schematic diagram for the partial unwinding of DNA staple strands caused extension. **a,** Schematic of digoxin antigen modified staple strand in Nat. Nanotech. 2019. The magenta circle represents digoxin, with a radius of ~1 nm. The antigen is connected to the short chain by 2 nt linker, along with partial unwinding, which might cause an extension of over 2 nm. **b**, Schematic of digoxin antigen directly modified staple strand in our work, without linker strand the extension is reduced. **c**, Possible mechanism for Cis-Form and Trans-Form epitope sites induced different extension degrees. The double Tran-Form epitope sites design on the right is likely to induce a maximum extension length that over 2 nm on a single site, and thus larger than ± 4 nm for a two-site distance. Which implies 16 nm distance could recruit 10 nm state IgGs.

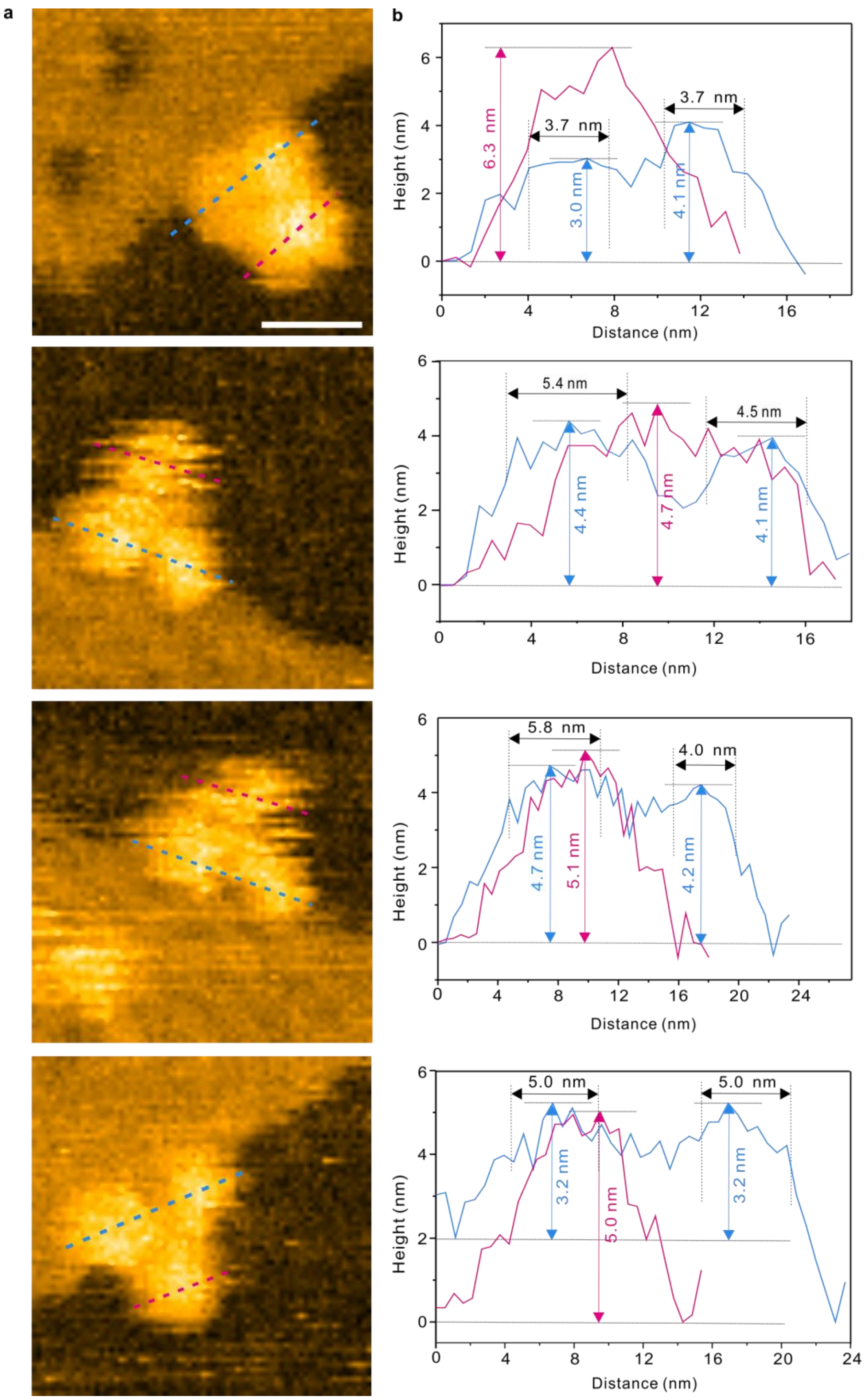

Supplementary Figure 3. Measured dimension of three domains of IgGs. a, HS-AFM images of four representative IgGs bound to DOEs (lateral distances: 5 nm, 8 nm, 10 nm, and 16 nm, respectively). Scale bar, 10 nm. **b,** Cross-sectional profiles of IgGs along their Fab domains: height (blue arrows) and width (black arrows). Cross-sectional profile of IgGs along their Fc domains: height (magenta arrows). Source data are provided as a Source Data file.

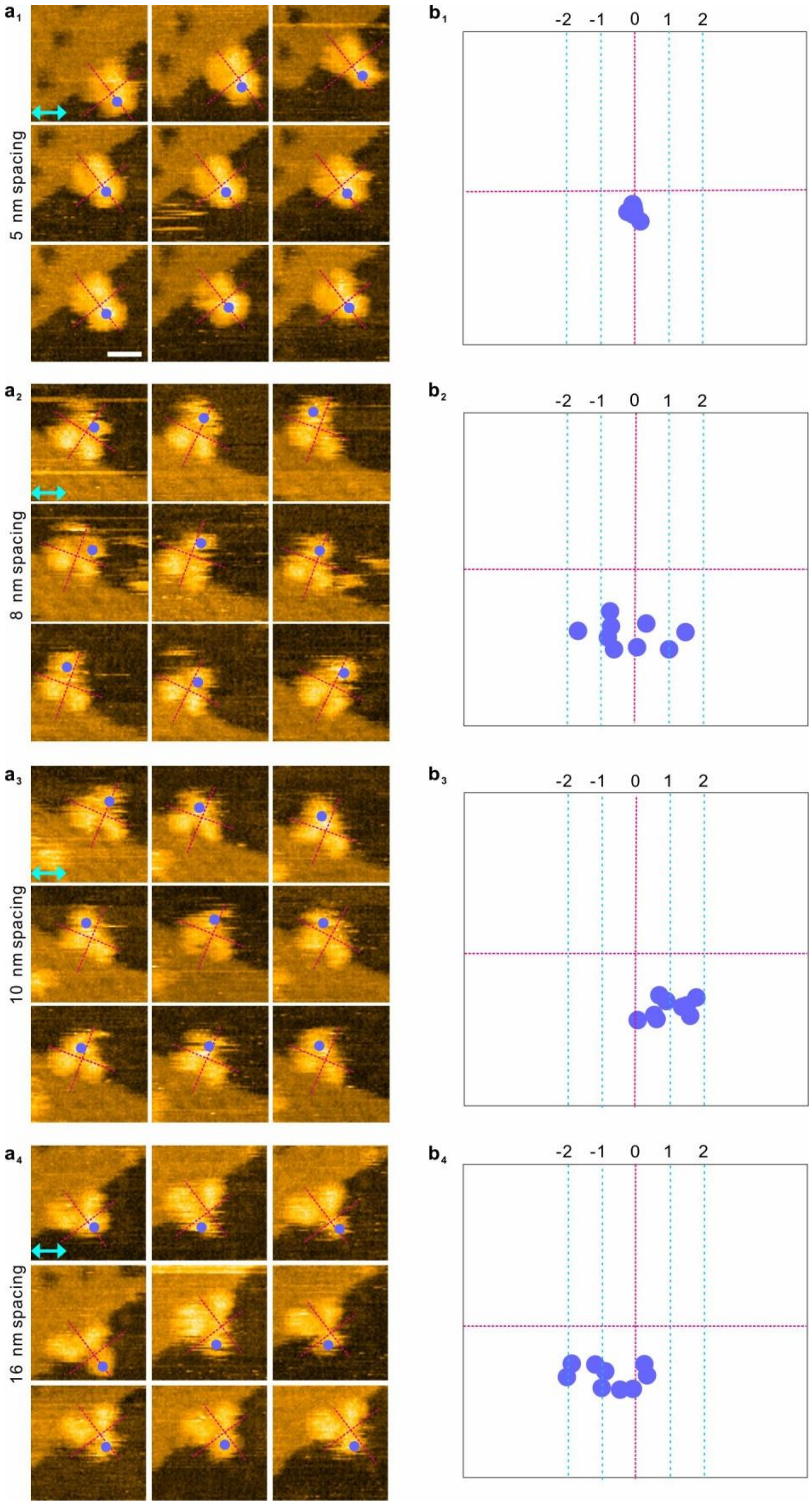

Supplementary Figure 4. Typical HS-AFM images of IgGs with wagging motion in the Fc domain. **a_1-a_4,** AFM images of IgGs. The barycenter of each Fc domain is marked by a blue dot; the center of the magenta crux represents the center of the three domains of IgG; one axis is parallel to the side of DNA origami bond by IgGs. (lateral distances: 5 nm, 8 nm, 10 nm, and 16 nm, respectively) **b_1-b_4,** Collected dot positions in **a_1-a_4**. Arrows indicate the scan directions. Scale bar, 10 nm.

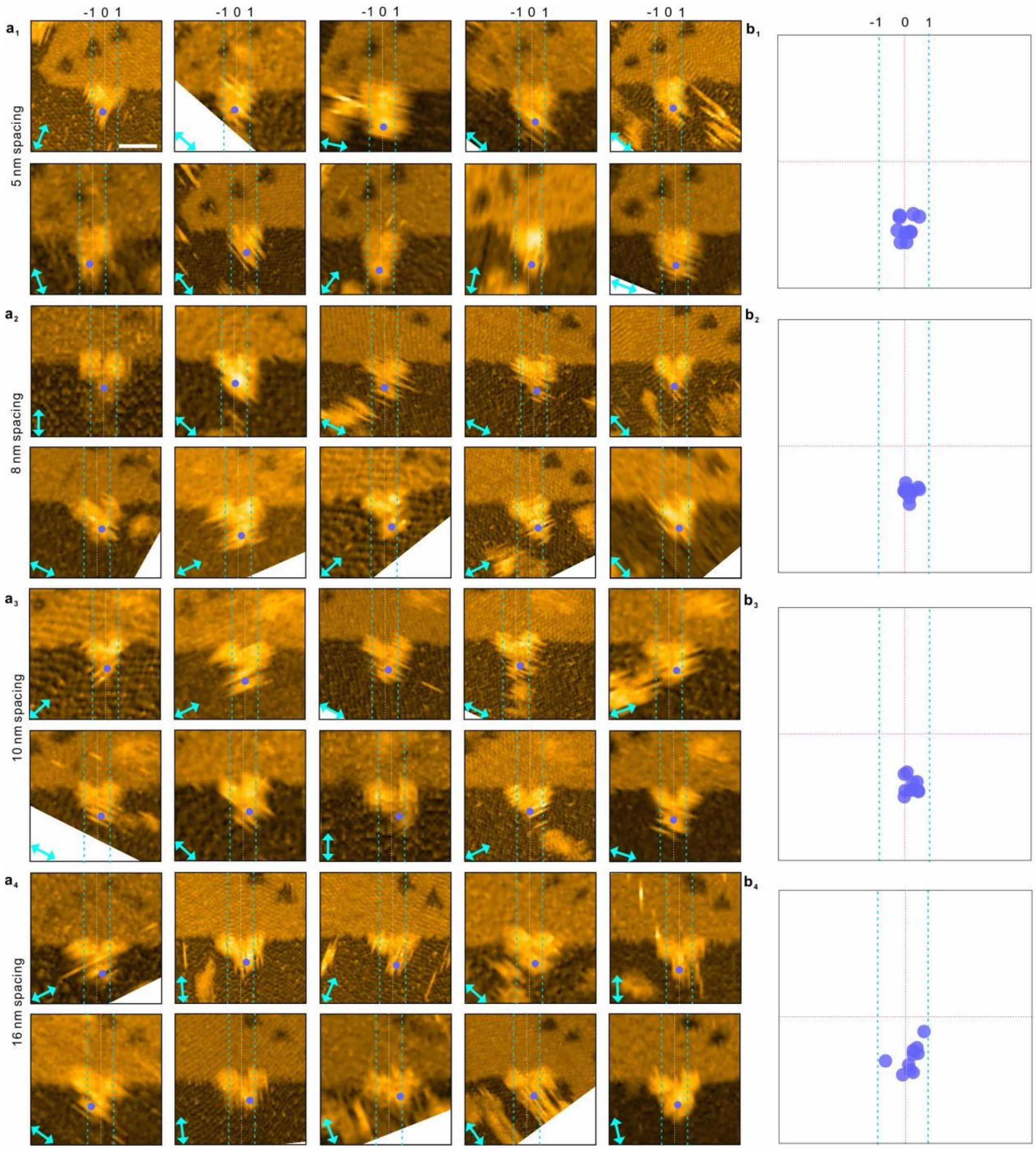

Supplementary Figure 5. HS-AFM images of Fc wagging motion in twenty two independent experiments. a_1-a_4, The Fc wagging motion of lateral distances 5 nm, 8 nm, 10 nm, and 16 nm respectively. The barycenter of each Fc domain is marked by a blue dot; the white dashed line represents the symmetry axis of the antibody be used as the zero position, the axis is perpendicular to the side of DNA origami bond by IgGs. The green dash lines are ± 1 nm from symmetry axis of the antibody. Arrows indicate the scan directions. Scale bar, 10 nm. **b_1-b_4,** Collected dot positions in **a_1-a_4**.

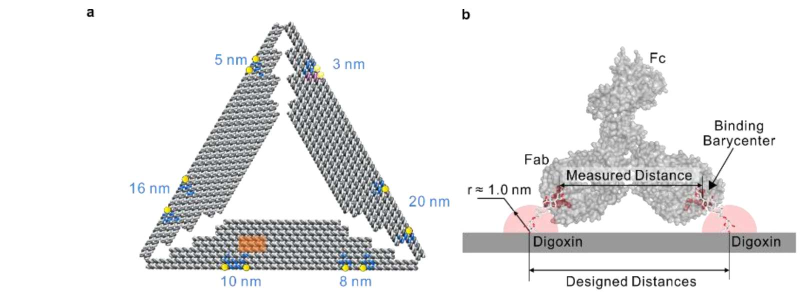

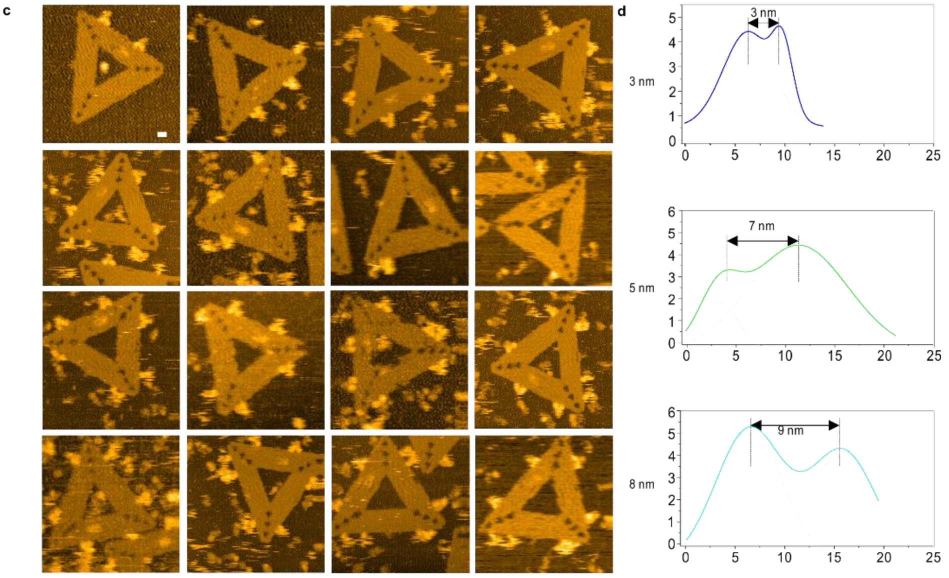

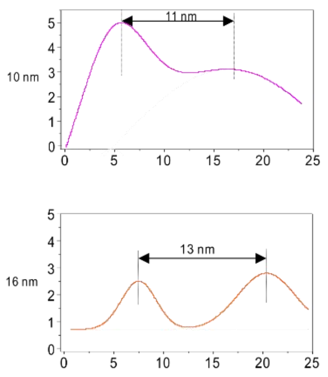

e

Designed Distance	3 nm	5 nm	8 nm	10 nm	16 nm
Measured Distance	3 nm	7 nm	9 nm	11 nm	12 nm
	5 nm	4 nm	8 nm	9 nm	10 nm
	5 nm	6 nm	9 nm	10 nm	13 nm
	5 nm	6 nm	7 nm	9 nm	10 nm
	5 nm	7 nm	8 nm	11 nm	13 nm
	5 nm	5 nm	8 nm	9 nm	11 nm
	6 nm	7 nm	10 nm	10 nm	11 nm
	4 nm	6 nm	8 nm	9 nm	12 nm
	3 nm	5 nm	7 nm	9 nm	11 nm
	5 nm	7 nm	7 nm	10 nm	10 nm
Mean Distance	4.6 nm	6.0 nm	8.1 nm	9.7nm	11.3 nm
Standard Deviation	1.0 nm	1.0 nm	1.0 nm	0.8 nm	1.2 nm

Supplementary Figure 6. Measured distances of two binding Fab in a single IgG molecule. a, Schematic design of the various distances of digoxin pairs on DNA origami. **b,** Schematic illustration of the measured Fab–Fab lengths along the two Fab barycenters and designed epitope of DOE. **c,** PeakForce-AFM images of IgGs bound on DOEs absorbed on a mica surface. **d,** Representative sectional profiles of measured binding distances of two Fabs in a single IgG molecule. Scale bar, 10 nm. **e,** The measured statistical data of the Fabs in IgGs (4.6 ± 1.0 nm, 6.0 ± 1.0 nm, 8.1 ± 1.0 nm, 9.7 ± 0.8 nm, and 11.3 ± 1.2 nm) and designed digoxin distances (3 nm, 5 nm, 8 nm, 10 nm, and 16 nm. The measured distance of two Fragments in a single IgG molecule was obtained by fitting cross-section profiles using the Gaussian model. Source data are provided as a Source Data file.

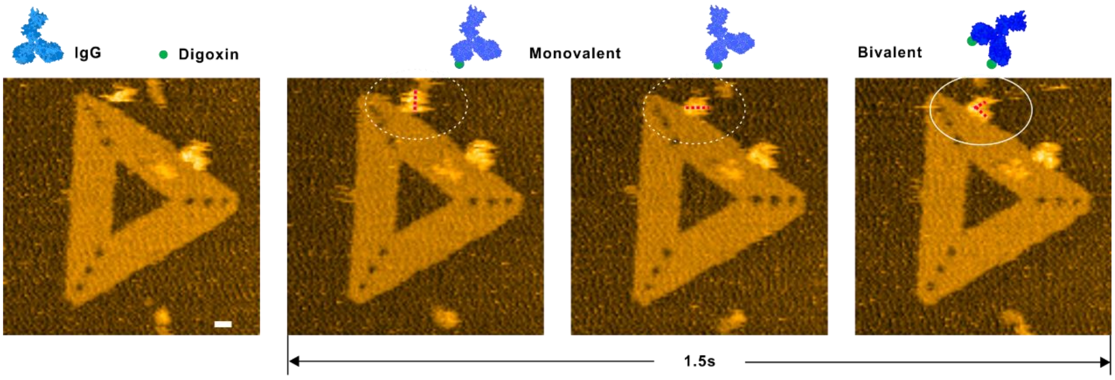

Supplementary Figure 7 (1). HS-AFM images recording the intermediate conformations of IgGs during the binding process from monovalent (dashed circles) to bivalent (solid circle) binding on a single DOE. The magenta lines indicate the orientation, position, and conformation of the same single IgG molecule. Scale bar, 10 nm.

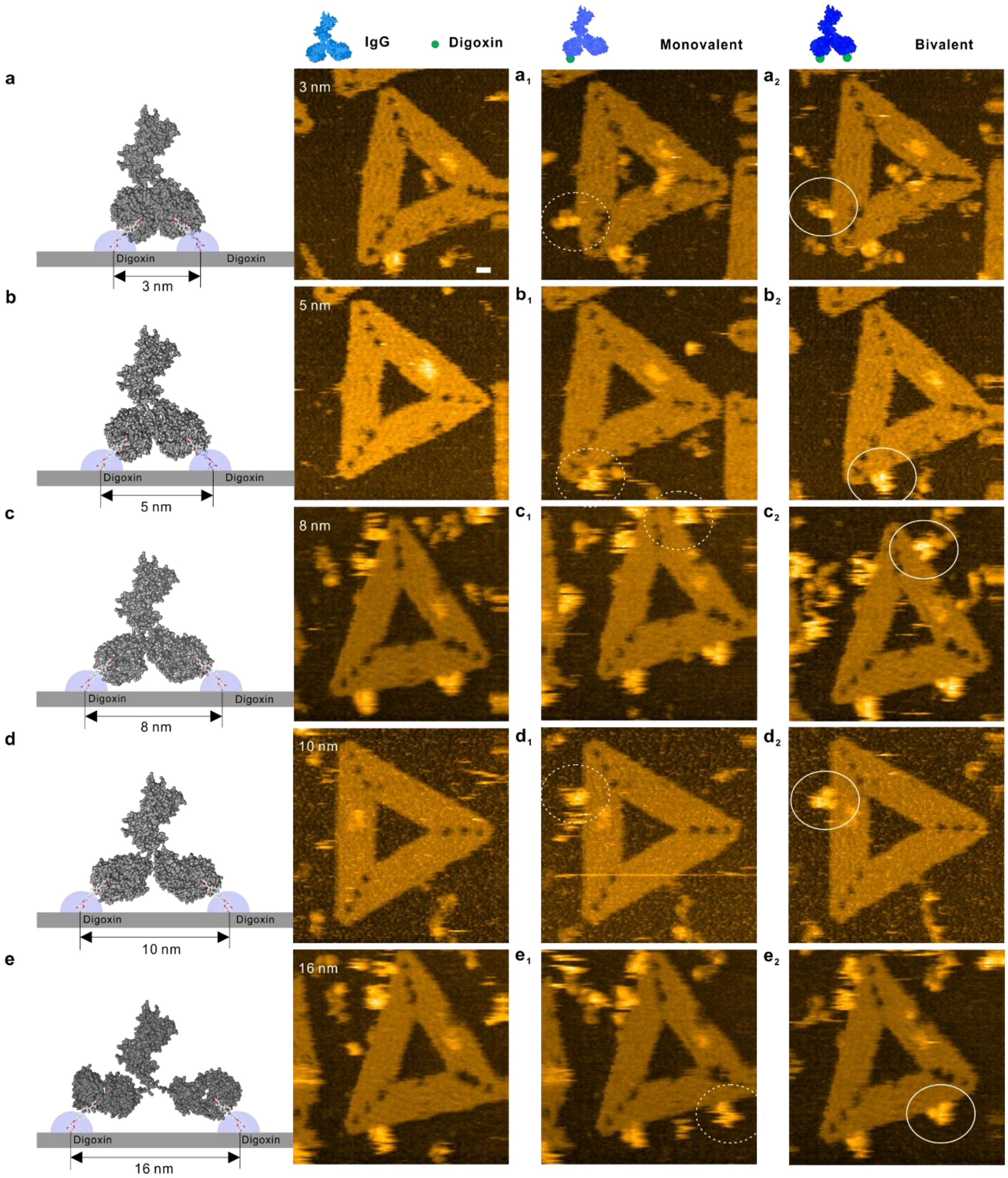

Supplementary Figure 7 (2). HS-AFM images of the monovalent (dashed circles) to bivalent binding (solid circles) steps during digoxin-IgG complex formation for various lateral distances (3 nm, 5 nm, 8 nm, 10 nm, and 16 nm) on DOEs. Scale bar, 10 nm.

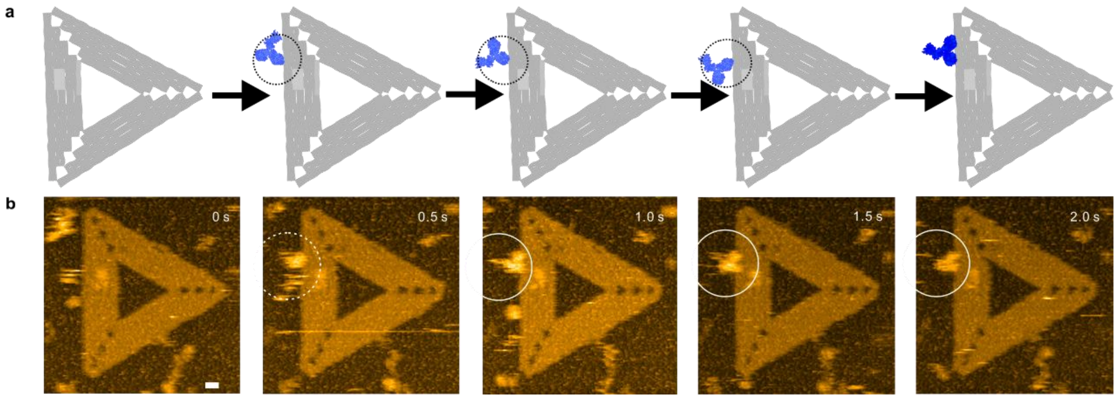

Supplementary Figure 8. Dynamic process of digoxin-IgG complex formation in a lateral distance of digoxin in 10 nm. a, Schematic diagram representing the dynamic process of a single IgG molecule binding to its cognate epitopes. **b,** HS-AFM images of the dynamic process of digoxin-IgG complex formation. Monovalent binding (white dash circle), bivalent binding (white solid circle). Scale bar, 10 nm.

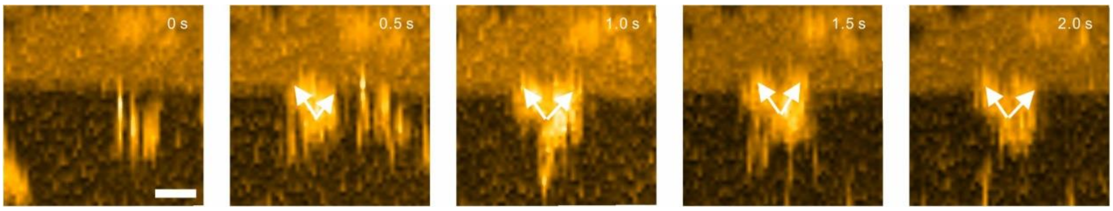

Supplementary Figure 9. Enlarged HS-AFM images of dynamic conformations and orientations of a single IgG molecule during digoxin-IgG complex formation for the lateral distance of digoxin in 10 nm (white arrows indicate the projection of the two Fabs of IgGs). Scale bar, 10 nm.

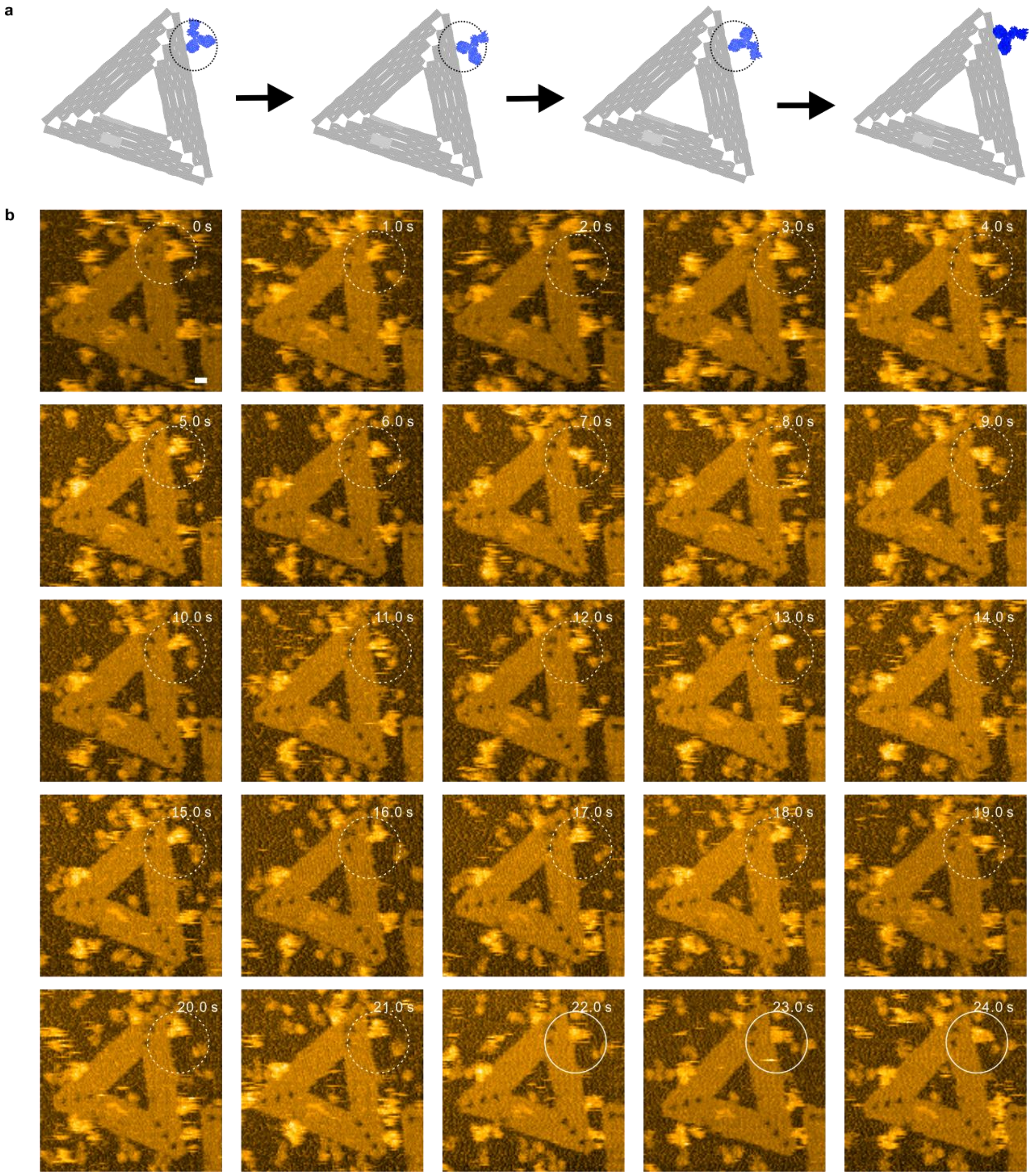

Supplementary Figure 10. Dynamic process of digoxin-IgG complex formation in a lateral distance of digoxin in 3 nm. a, Schematic diagram representing the dynamic process of a single IgG binding to its cognate epitopes. **b,** HS-AFM images of the dynamic process of digoxin-IgG complex formation. Monovalent binding (white dash circle), bivalent binding (white solid circle). Scale bar, 10 nm.

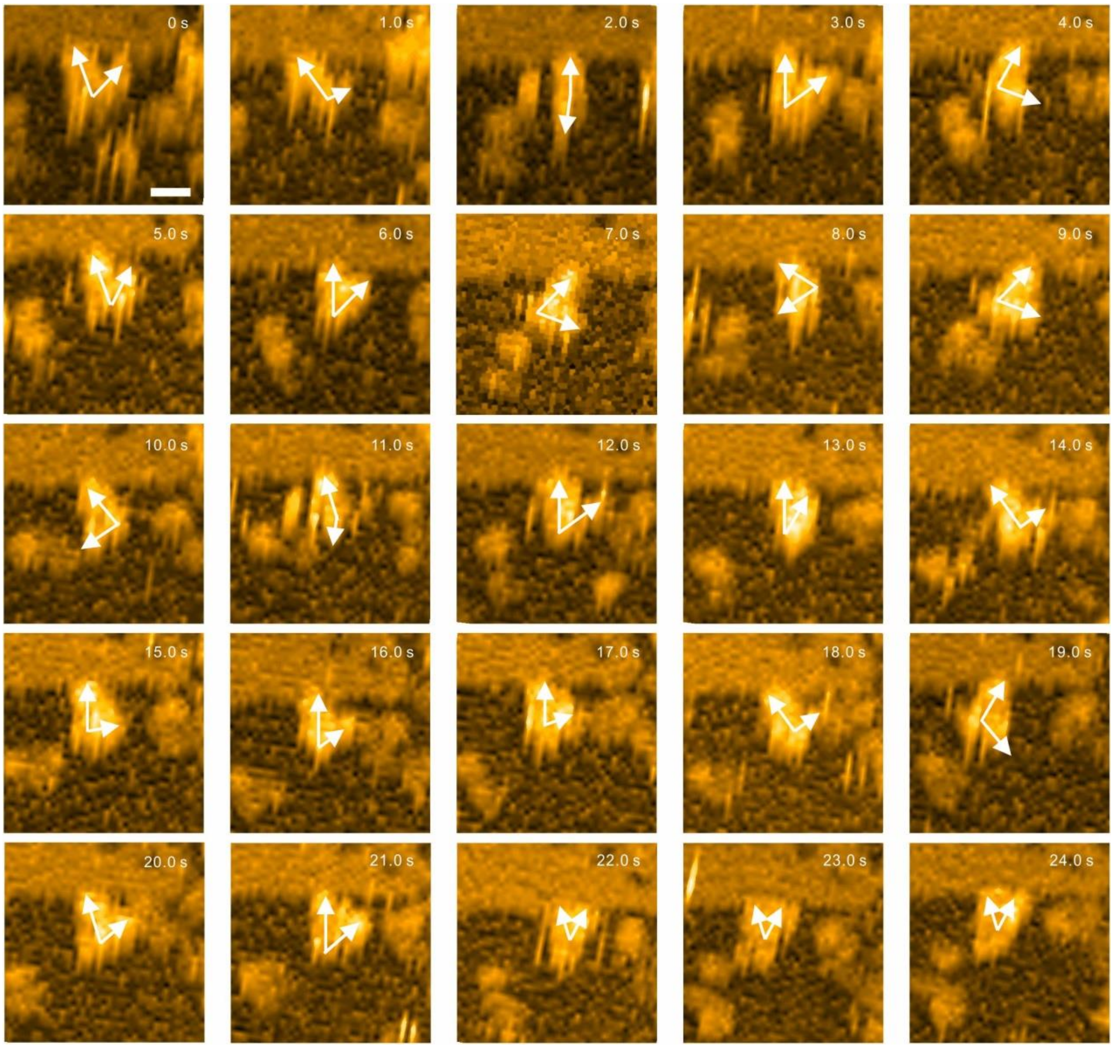

Supplementary Figure 11. Enlarged HS-AFM images of dynamic conformations and orientations of a single IgG molecule during digoxin-IgG complex formation for the lateral distance of digoxin in 3 nm (white arrows indicate the projection of the two Fabs of IgG). Scale bar, 10 nm.

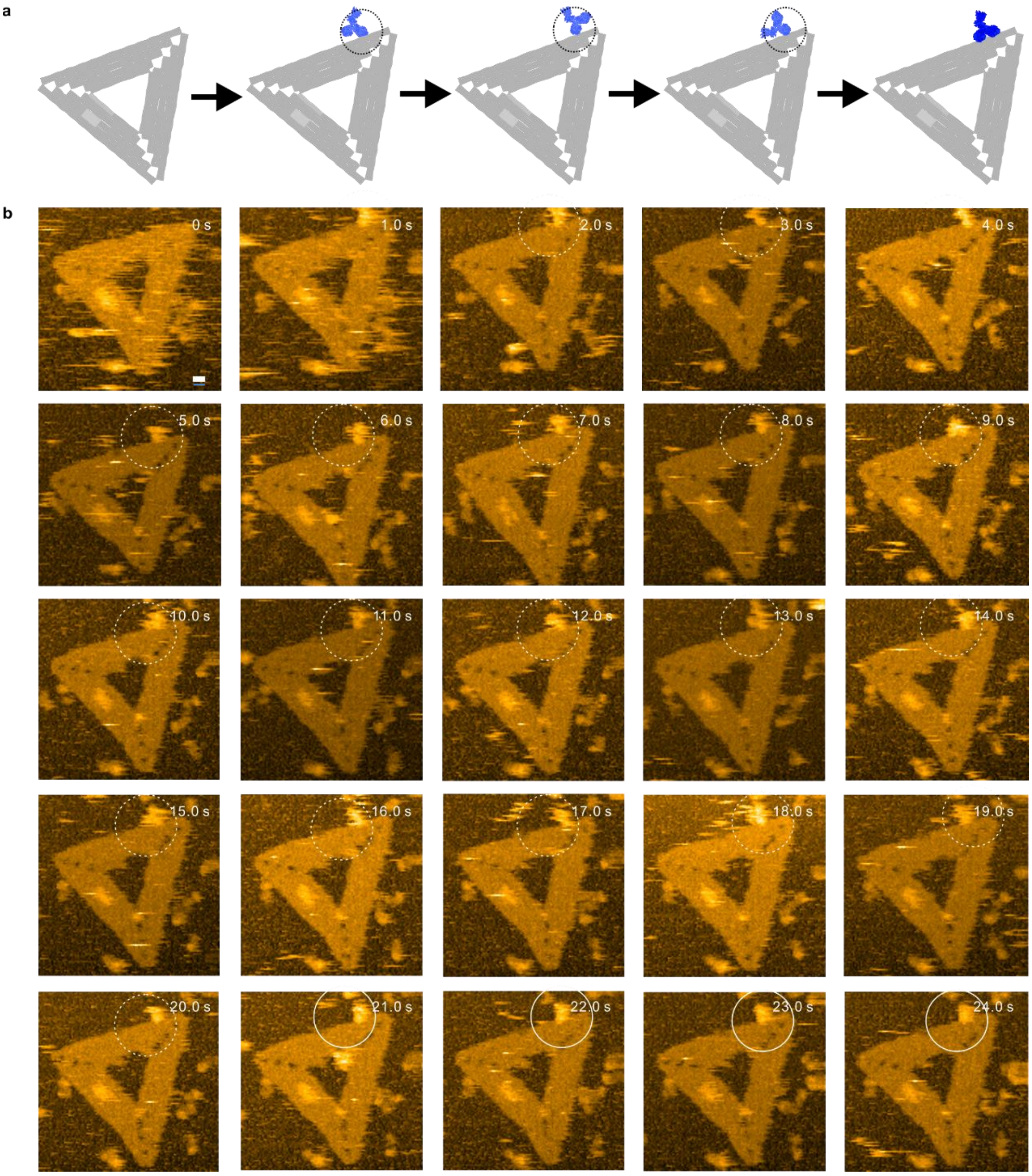

Supplementary Figure 12. Dynamic process of digoxin-IgG complex formation in a lateral distance of digoxin in 5 nm. a, Schematic diagram representing the dynamic process of a single IgG molecule binding to its cognate epitopes. **b,** HS-AFM images of the dynamic process of digoxin-IgG complex formation. Monovalent binding (white dash circles), bivalent binding (white solid circles). Scale bar, 10 nm.

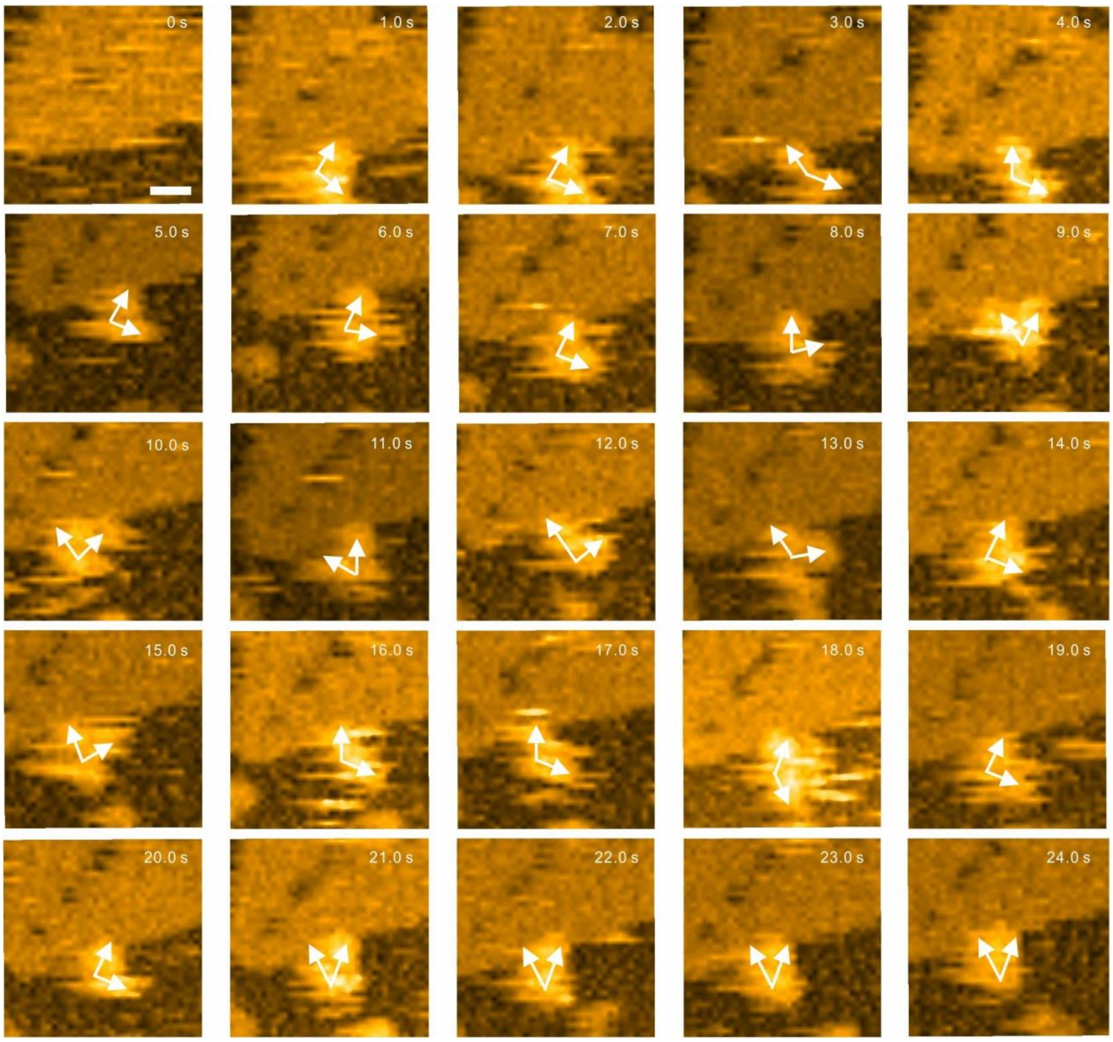

Supplementary Figure 13. Enlarged HS-AFM images of dynamic conformations and orientations of a single IgG molecule during digoxin-IgG complex formation for the lateral distance of digoxin in 5 nm (white arrows indicate the projection of the two Fabs of IgG). Scale bar, 10 nm.

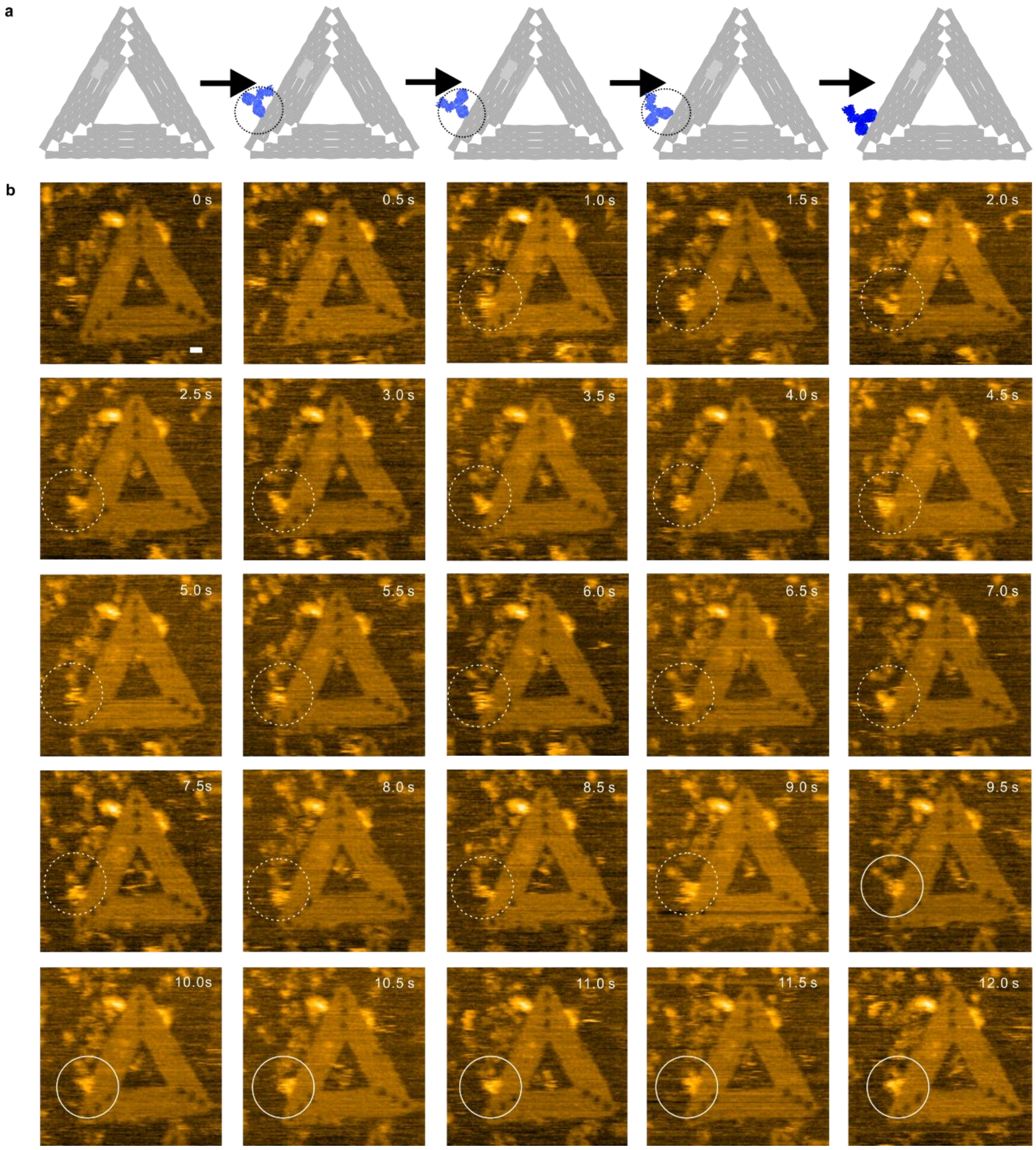

Supplementary Figure 14. Dynamic process of digoxin-IgG complex formation in a lateral distance of digoxin in 8 nm. a, Schematic diagram representing the dynamic process of single IgG binding to its cognate epitopes. **b,** HS-AFM images of the dynamic process of digoxin-IgG complex formation. Monovalent binding (white dash circles), bivalent binding (white solid circles). Scale bar, 10 nm.

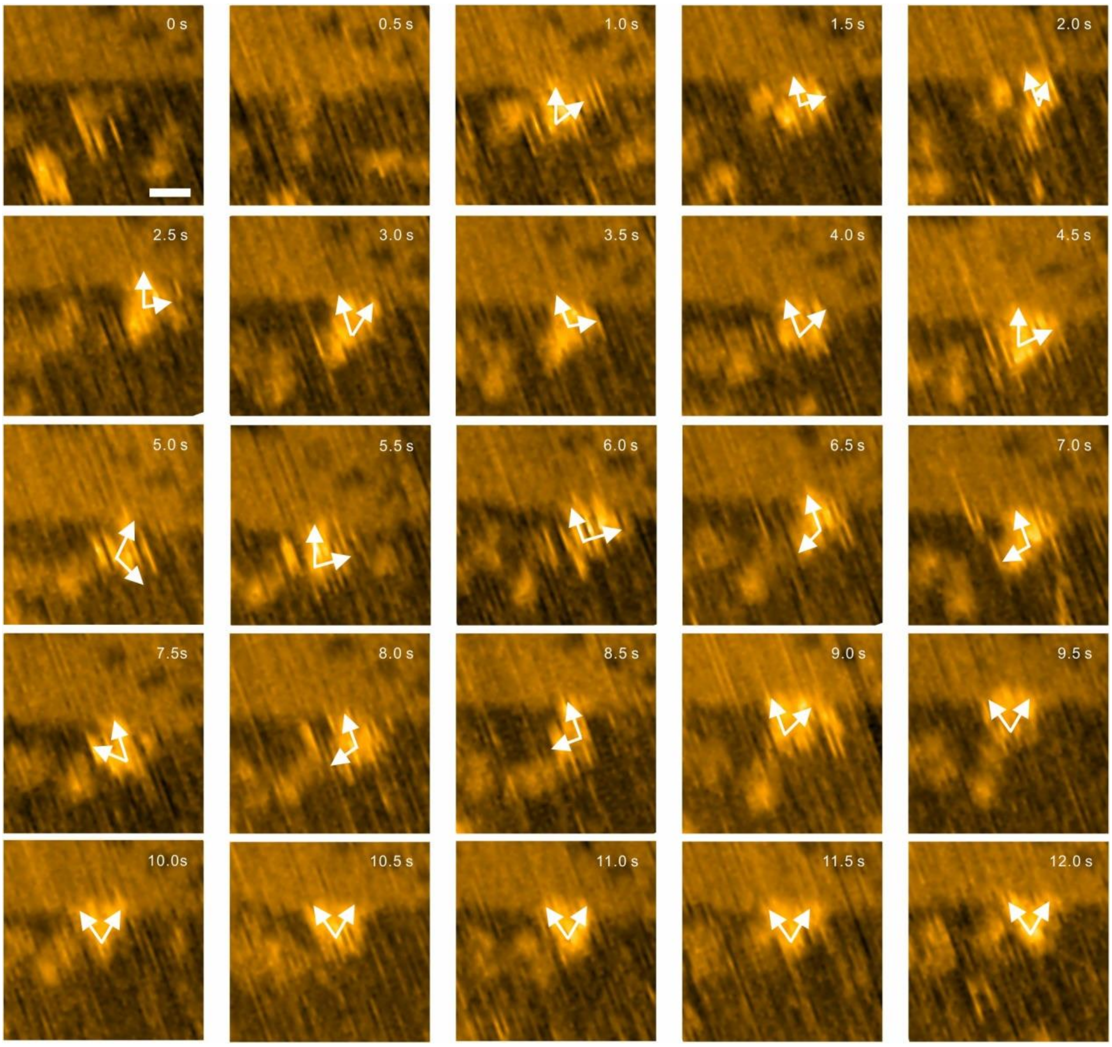

Supplementary Figure 15. Enlarged HS-AFM images of dynamic conformations and orientations of a single IgG molecule during digoxin-IgG complex formation for the lateral distance of digoxin in 8 nm (white arrows indicate the projection of the two Fabs). Scale bar, 10 nm.

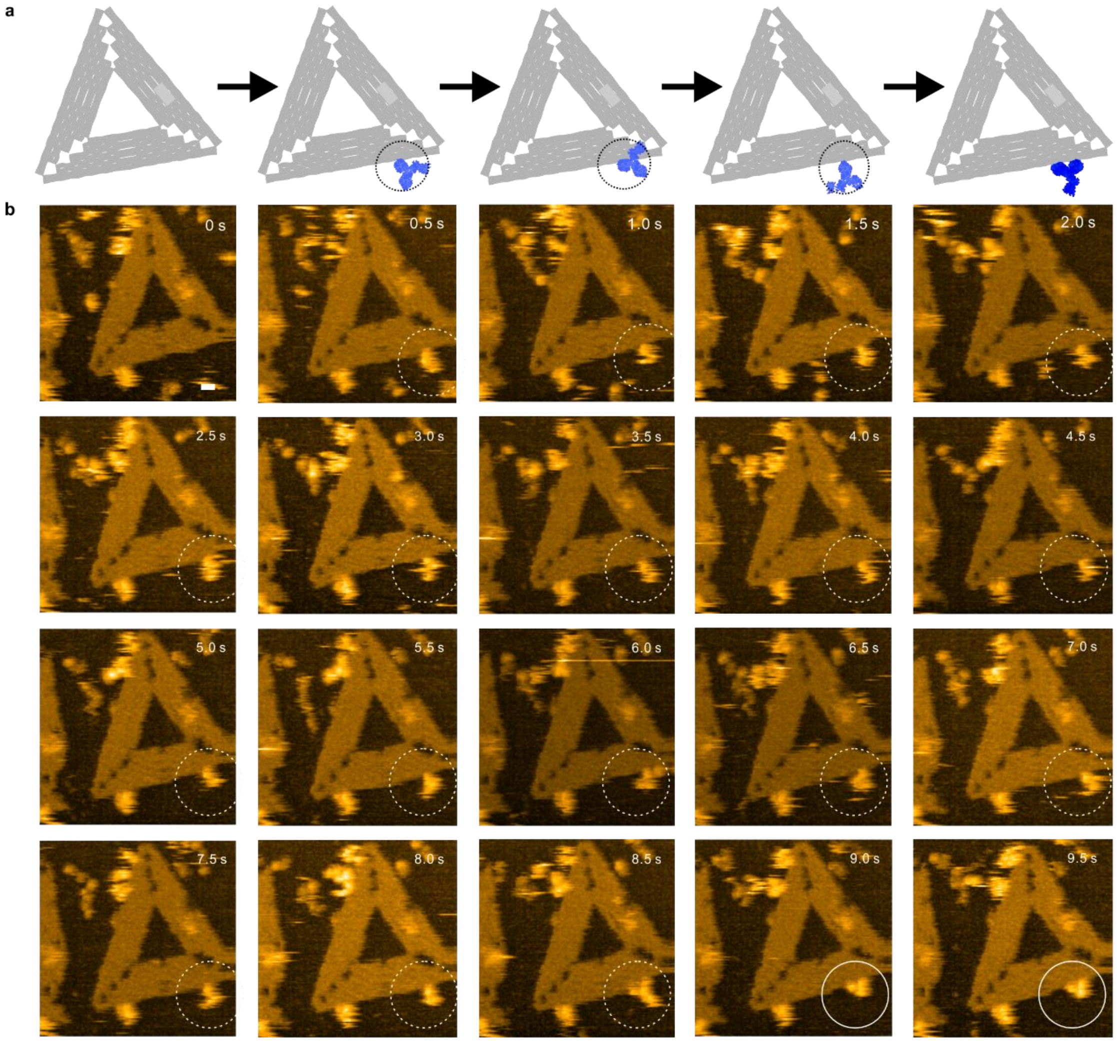

Supplementary Figure 16. Dynamic process of digoxin-IgG complex formation in a lateral distance of digoxin in 16 nm. a, Schematic diagram representing the dynamic process of single IgG binding to its cognate epitopes. **b,** HS-AFM images of the dynamic process of digoxin-IgG complex formation. Monovalent binding (white dash circles), bivalent binding (white solid circles). Scale bar, 10 nm.

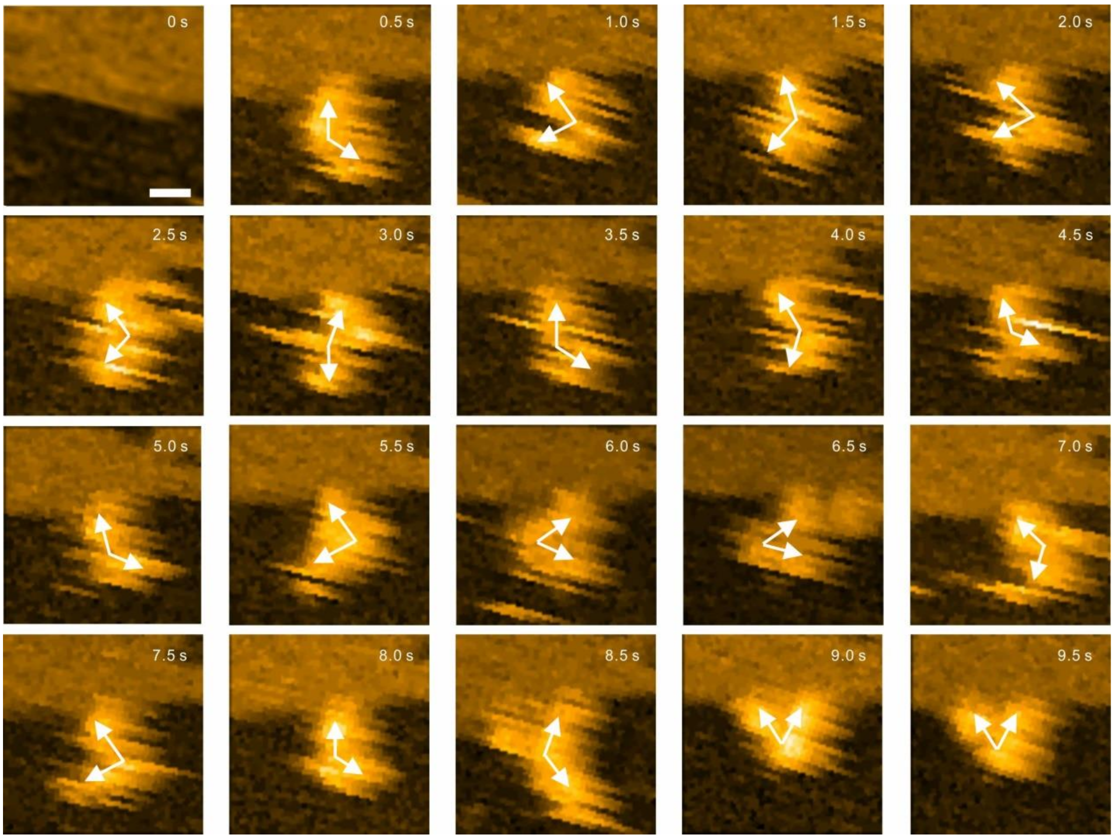

Supplementary Figure 17. Enlarged HS-AFM images of dynamic conformations and orientations of a single IgG during digoxin-IgG complex formation for the lateral distance of digoxin in 16 nm (white arrows indicate the projection of the two Fabs of IgG). Scale bar, 10 nm.

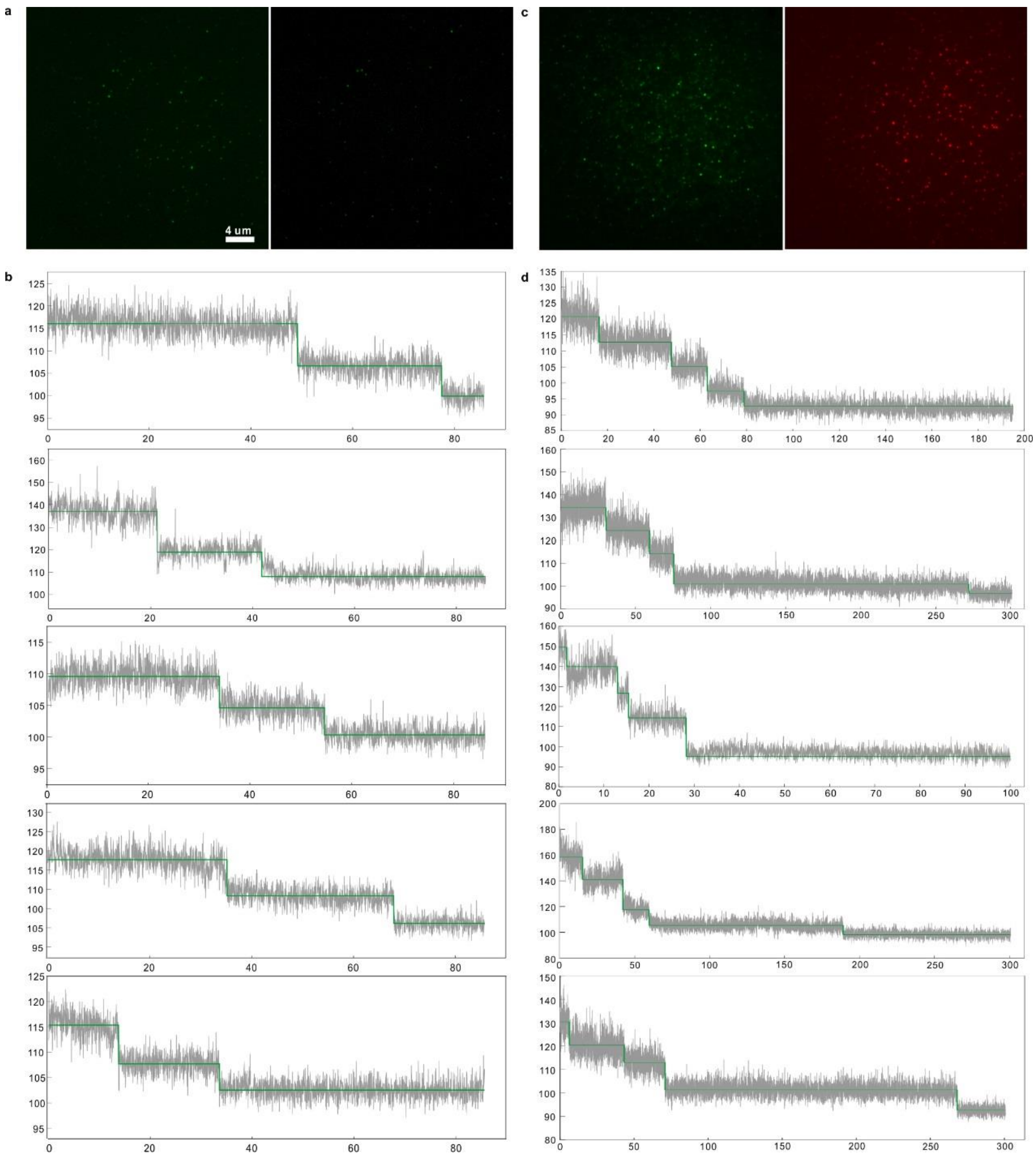

Supplementary Figure 18. smFRET signals for the lateral distance of digoxin in 10 nm of DOEs. a, Fluorescence images of the photobleaching on DOEs labeled with ATTO 550 when excited by 561 nm laser for 0 sec (left) and 90 sec (right). Scale bar, 4 um. **b,** The trajectory and hidden Markov model fit of photobleaching. **c,** Fluorescence images before and after adding antibodies labeled with Alexa 647 (from left to right) . The green and red channels are excited by 561 and 647 nm lasers, respectively. These images are analyzed for co-localization. **d,** Representative smFRET time trajectory of stable binding of two digoxin molecules on DOEs in the presence of IgGs. Source data are provided as a Source Data file.

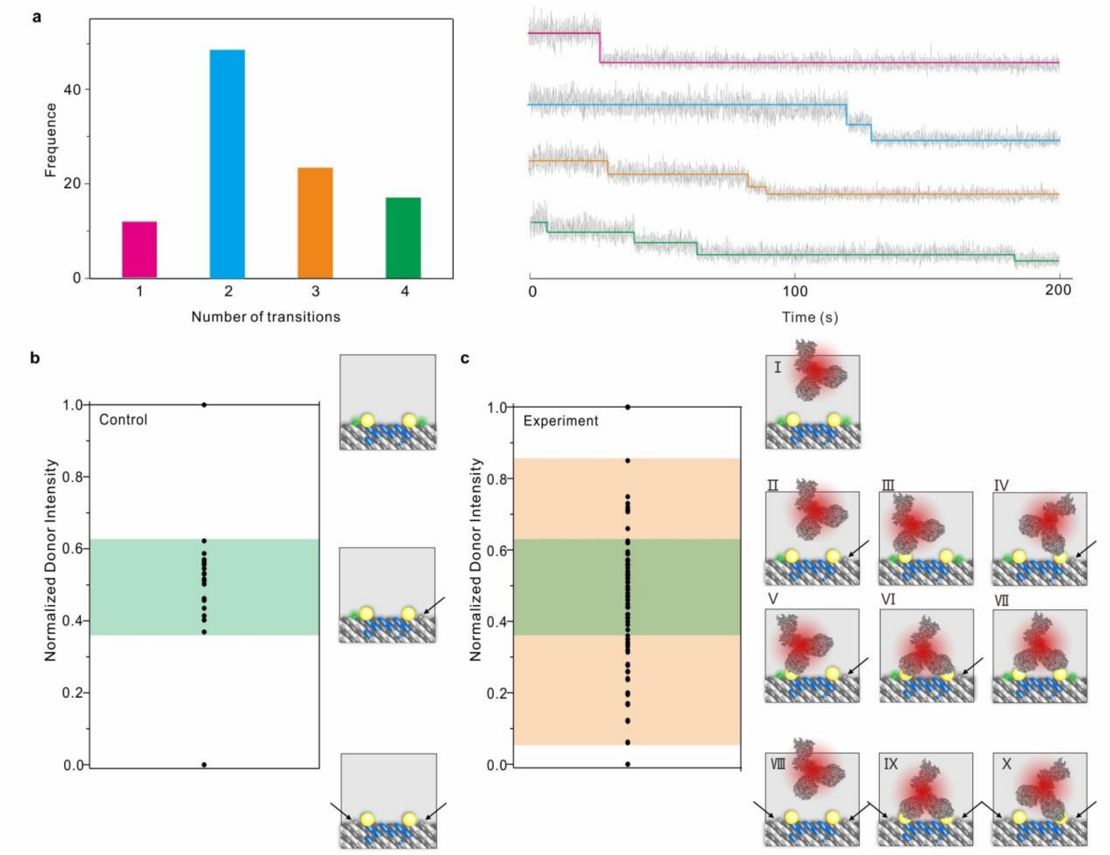

Supplementary Figure 19. Analysis of FRET data. a, Distribution of the attenuation number of fluorescent intensities of ATTO 550-DNA-origami after addition of Alexa 647-IgG. When the fluorescence intensity decreases by > 20%, comparing to the previous recorded fluorescence intensity, it was identified as an individual fluorescence event, in other word, one attenuation number. Note that only when attenuation number 4 indicates a complete binding event. **b,** In the absence of Alexa 647-IgG, the normalized fluorescence intensities can be clearly classed into three sets (1.00, 0.65–0.35, and 0.00), representing three states of two, one, and zero excited ATTO 550 molecules. **c,** In the presence of Alexa 647-IgG, the normalized fluorescence intensities were classed into three states (1.00, 0.85–0.10, and 0.00). First, 1.00, no binding, no bleaching (I). Second, 0.85-0.10: multiple possible states co-existed, including: no binding, one bleaching (II), monovalent binding, no bleaching (III), monovalent binding, close site bleaching (IV), monovalent binding, rear site bleaching (V), bivalent binding, one bleaching (VI), bivalent binding, no bleaching (VII). Third, 0.10-0: No energy transfer, double bleaching (VIII-X). Source data are provided as a Source Data file.

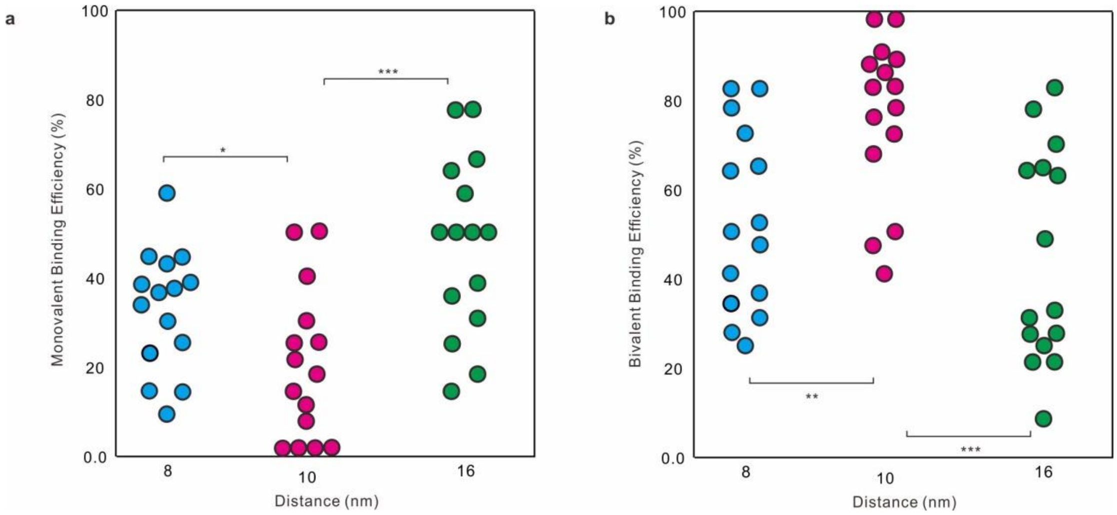

Supplementary Figure 20. Effect of epitope distance on IgG avidity. a, b Statistical analysis of binding efficiencies for monovalent (32.7 ± 13.5, 19.5 ± 17.4, 47.1 ± 19.9) and bivalent (60.1 ± 17.3, 80.6 ± 15.4, 53.0 ± 20.5) bonded IgG for the distance of 8 nm, 10 nm, and 16 nm (n = 166 DNA origamis). Data are presented as the means and standard deviations from three independent experiments. *p<0.05, **<0.01, ***<0.001, ANOVA. Source data are provided as a Source Data file.

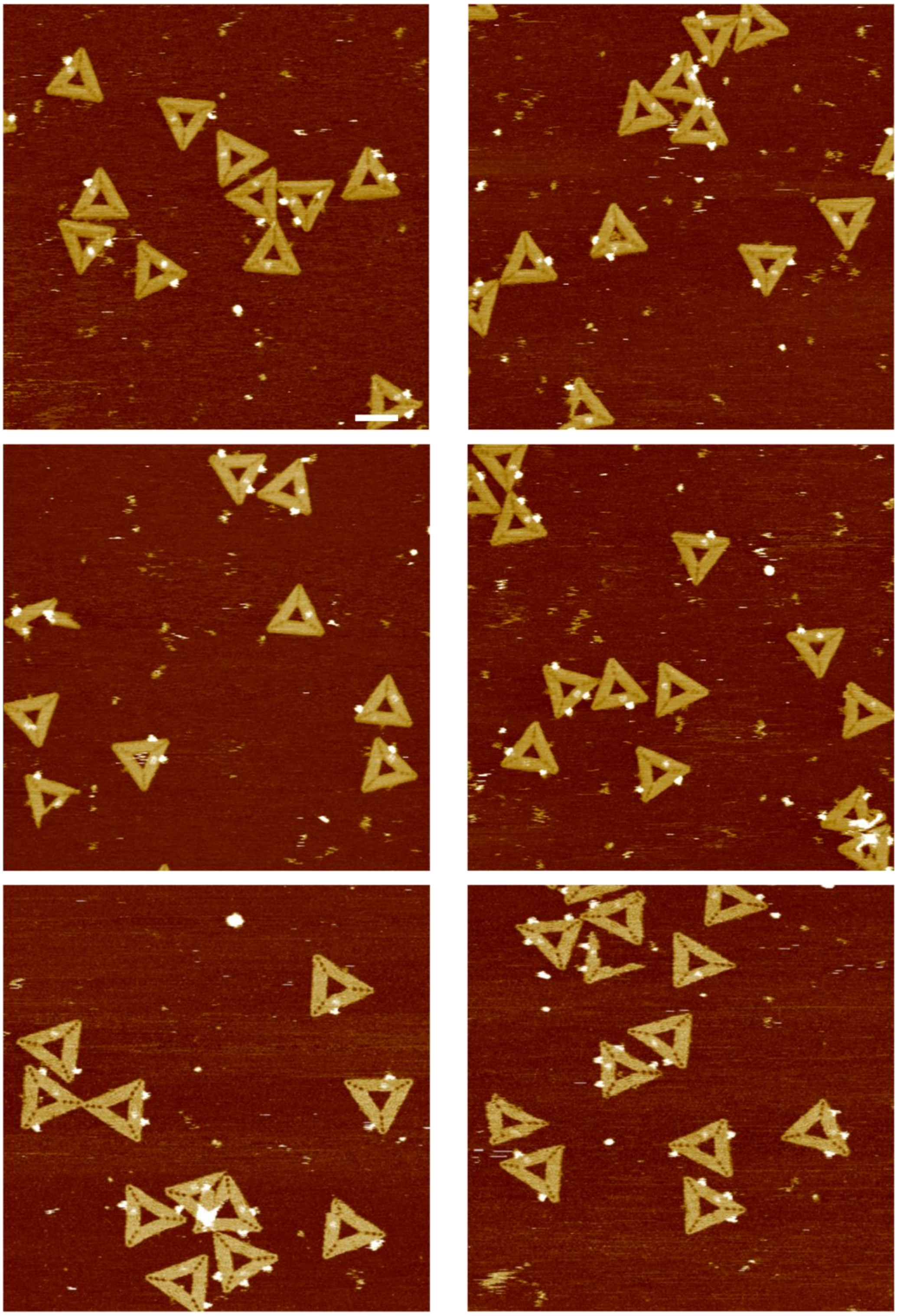

Supplementary Figure 21. PeakForce-AFM images of digoxin-IgG complexes in a final IgG dilution ratio of 1:6400. Scale bar, 100 nm.

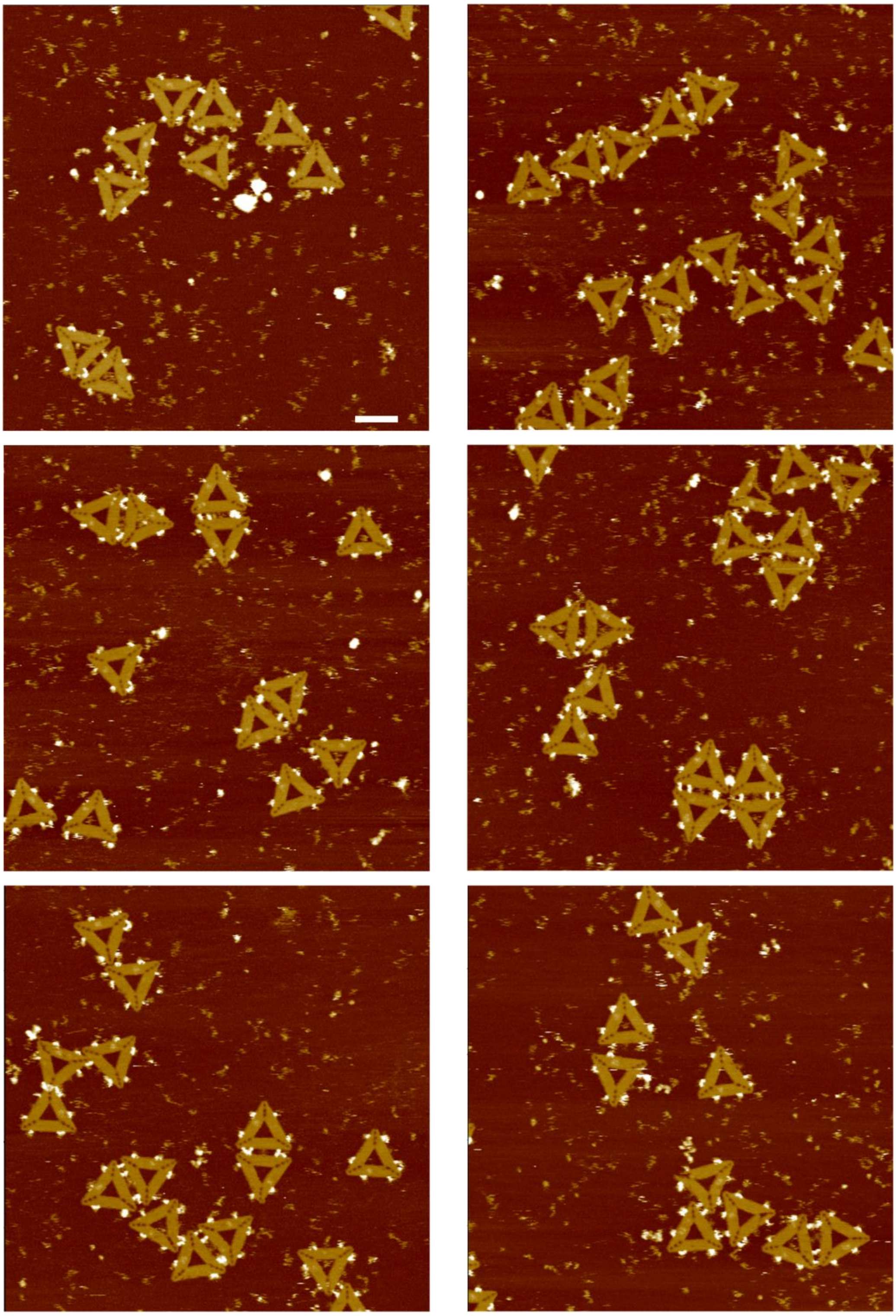

Supplementary Figure 22. PeakForce-AFM images of digoxin-IgG complexes in a final IgG dilution ratio of 1:3200. Scale bar, 100 nm.

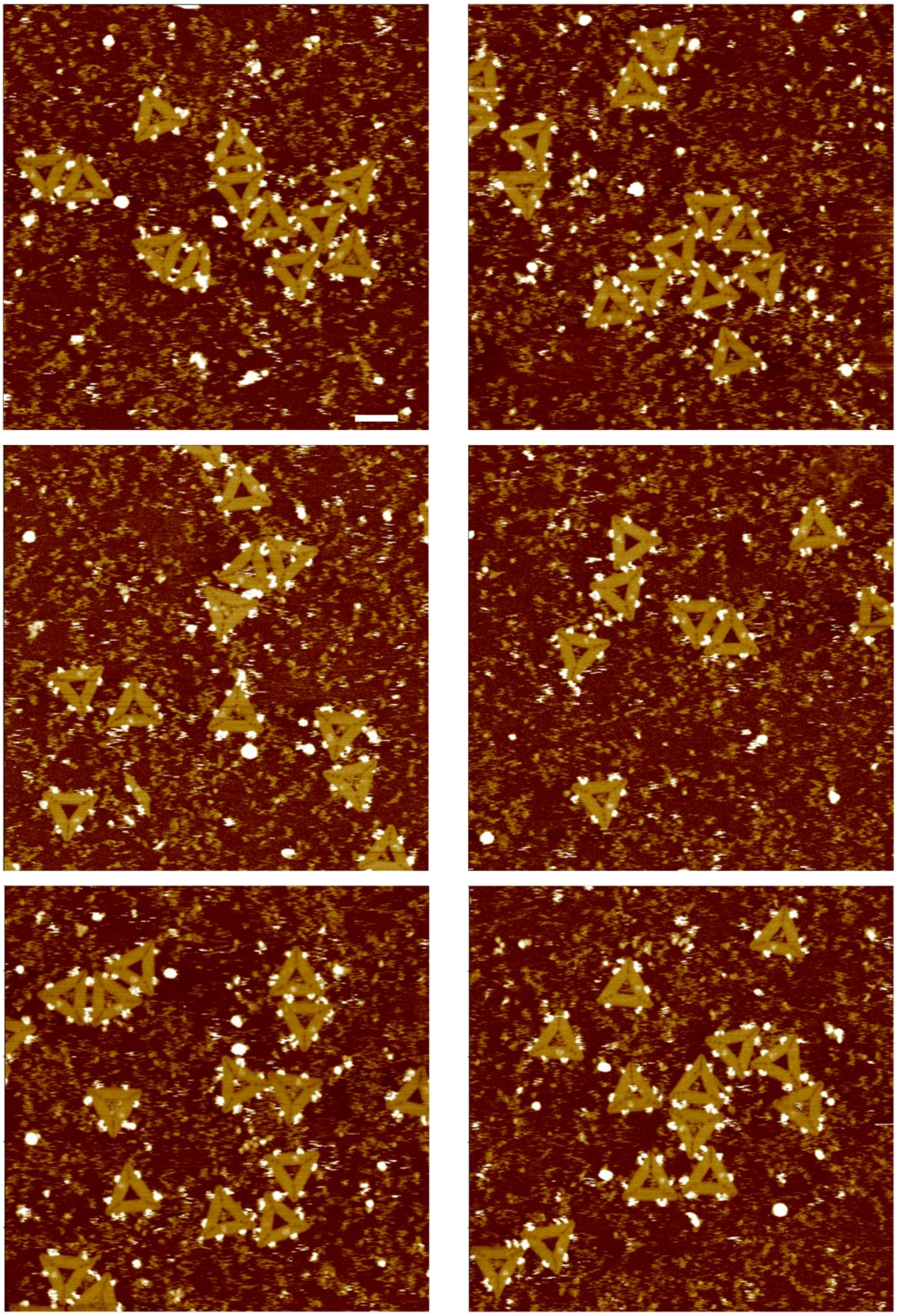

Supplementary Figure 23. **PeakForce-AFM images of digoxin-IgG complexes in a final IgG dilution ratio of 1:1600.** Scale bar, 100 nm.

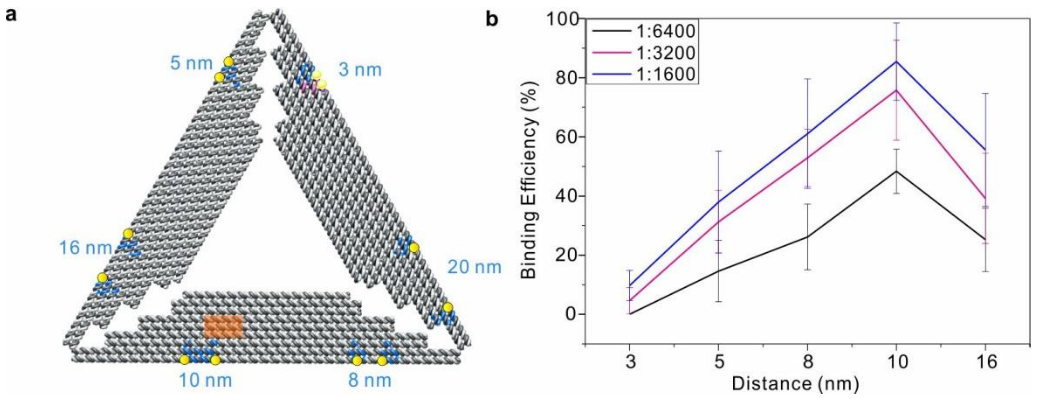

Supplementary Figure 24. Binding efficiency of digoxin-IgG complexes as a function of lateral distance of digoxin. a, Schematic diagram for the location of various designed distance. **b,** Binding efficiency of IgGs as a function of lateral distance of digoxin using different concentrations of antibodies. The error bars represent the standard deviation, which was calculated from three independent experiments for each concentration of IgGs, n = 252 DNA origamis. Source data are provided as a Source Data file.

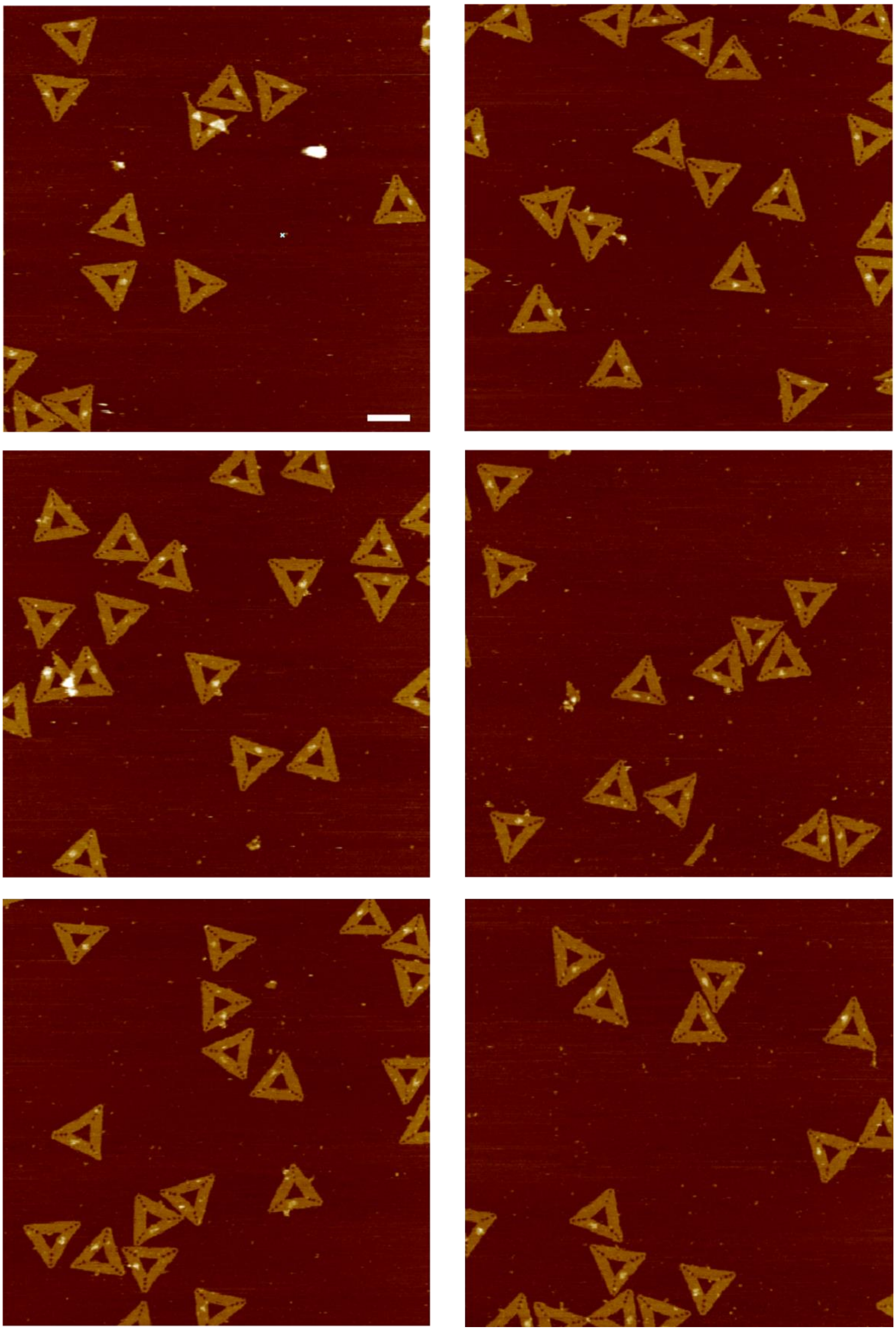

Supplementary Figure 25. PeakForce-AFM images of biotin-IgG complexes in a final IgG concentration of 30 nM. Scale bar, 100 nm.

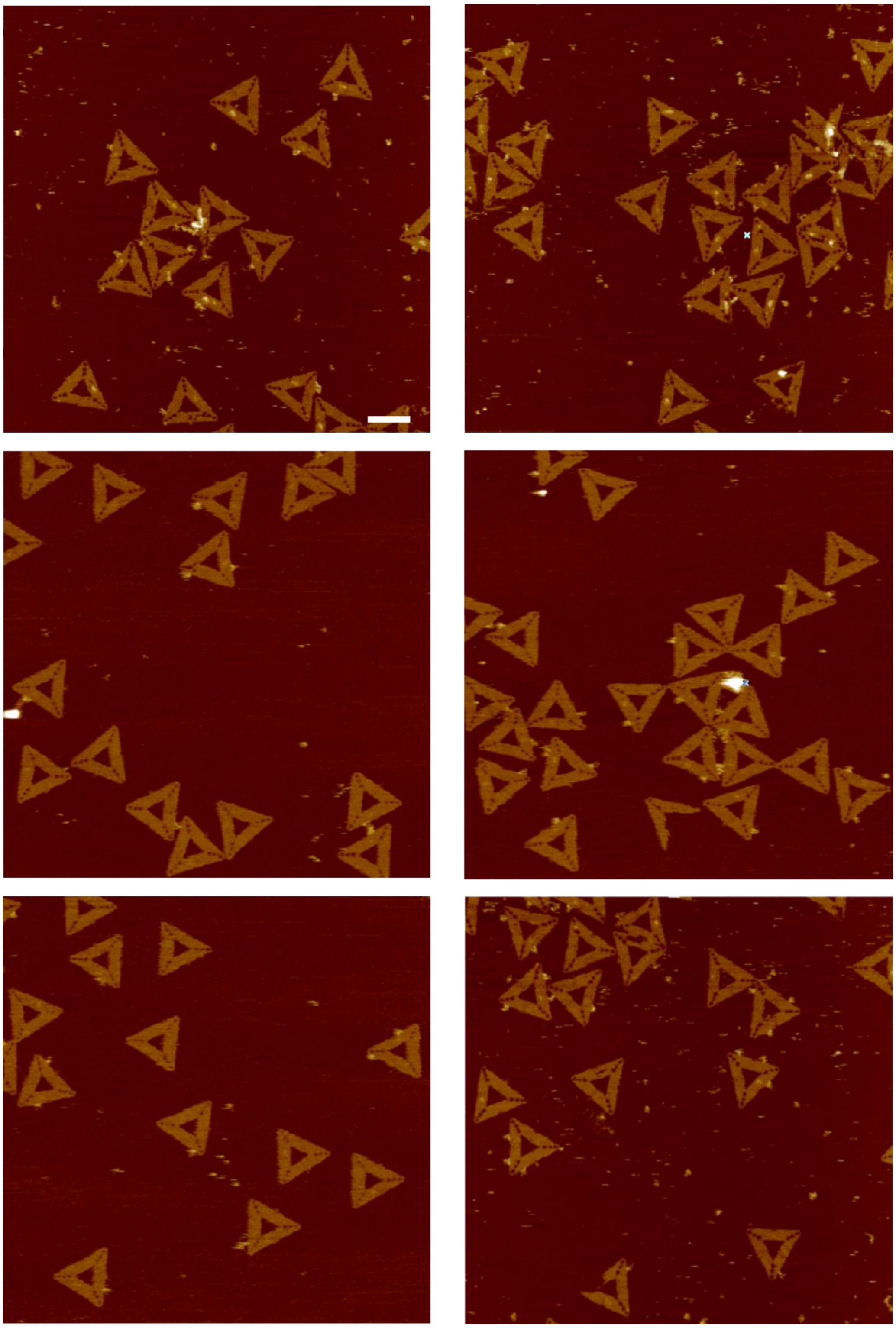

Supplementary Figure 26. PeakForce-AFM images of biotin-IgG complexes in a final IgG concentration of 60 nM. Scale bar, 100 nm.

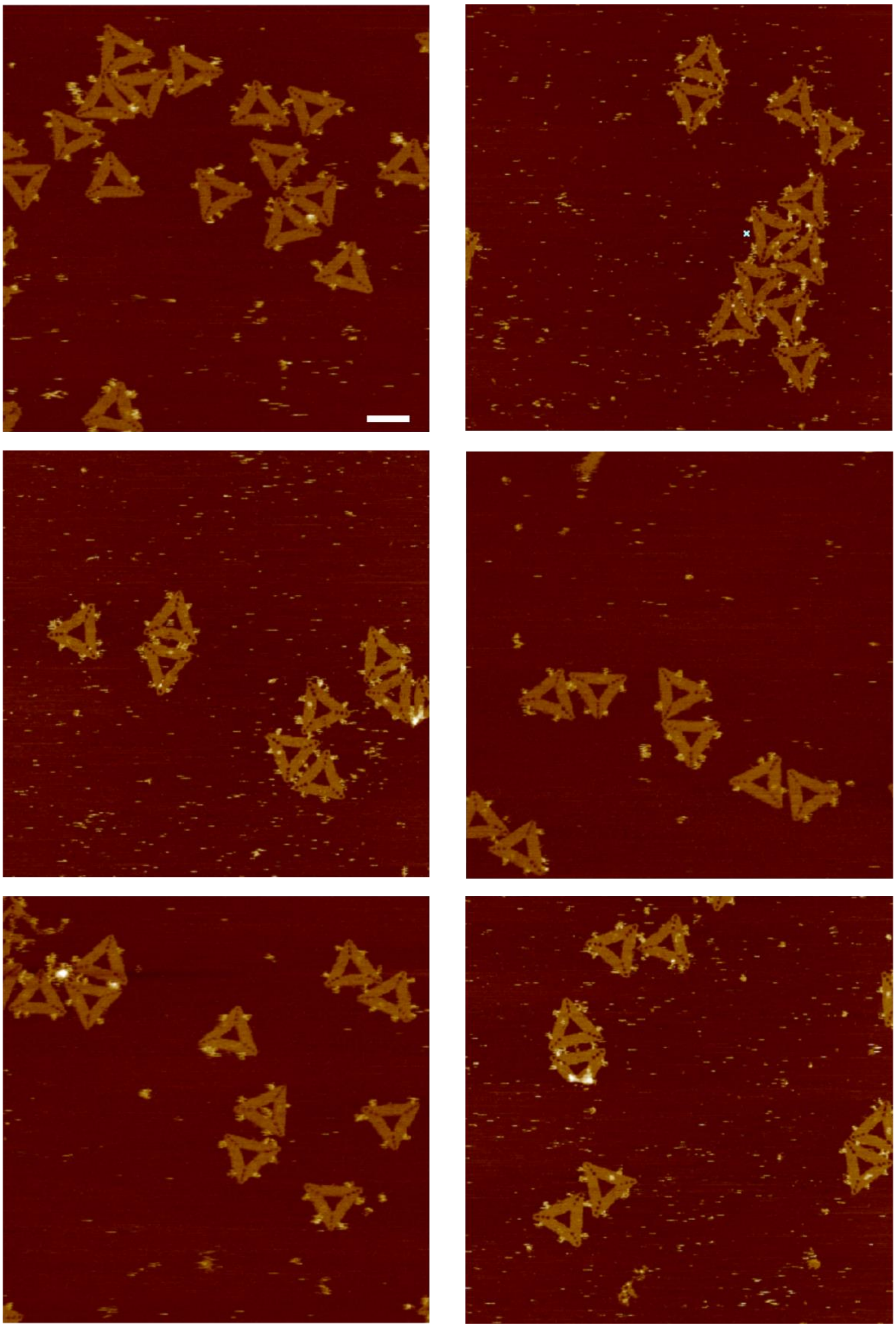

Supplementary Figure 27. PeakForce-AFM images of biotin-IgG complexes in a final IgG concentration of 90 nM. Scale bar, 100 nm.

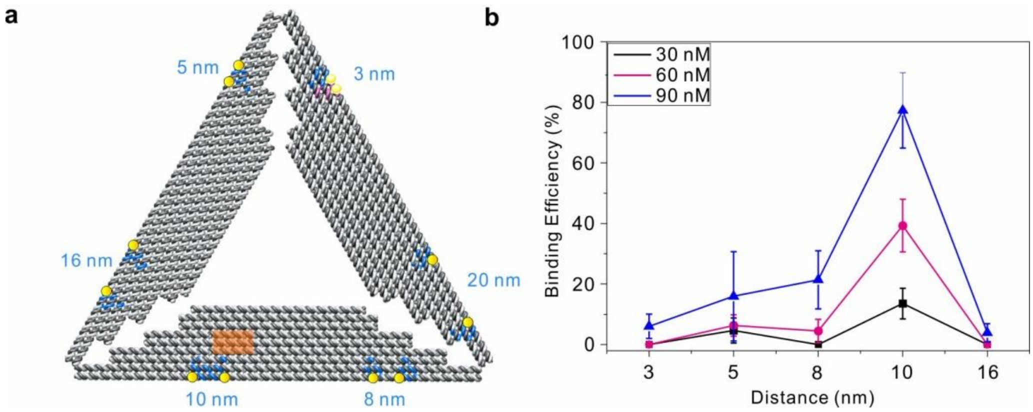

Supplementary Figure 28. Binding efficiency of biotin-IgG complexes as a function of lateral distance of biotin. a, Schematic diagram for the location of various designed distances. **b,** Binding efficiency of IgGs as a function of lateral distance of biotin using various concentrations of IgGs. The error bars represent the standard deviation, which was calculated from three independent experiments for each concentration of IgGs, n = 372 DNA origamis. Source data are provided as a Source Data file.

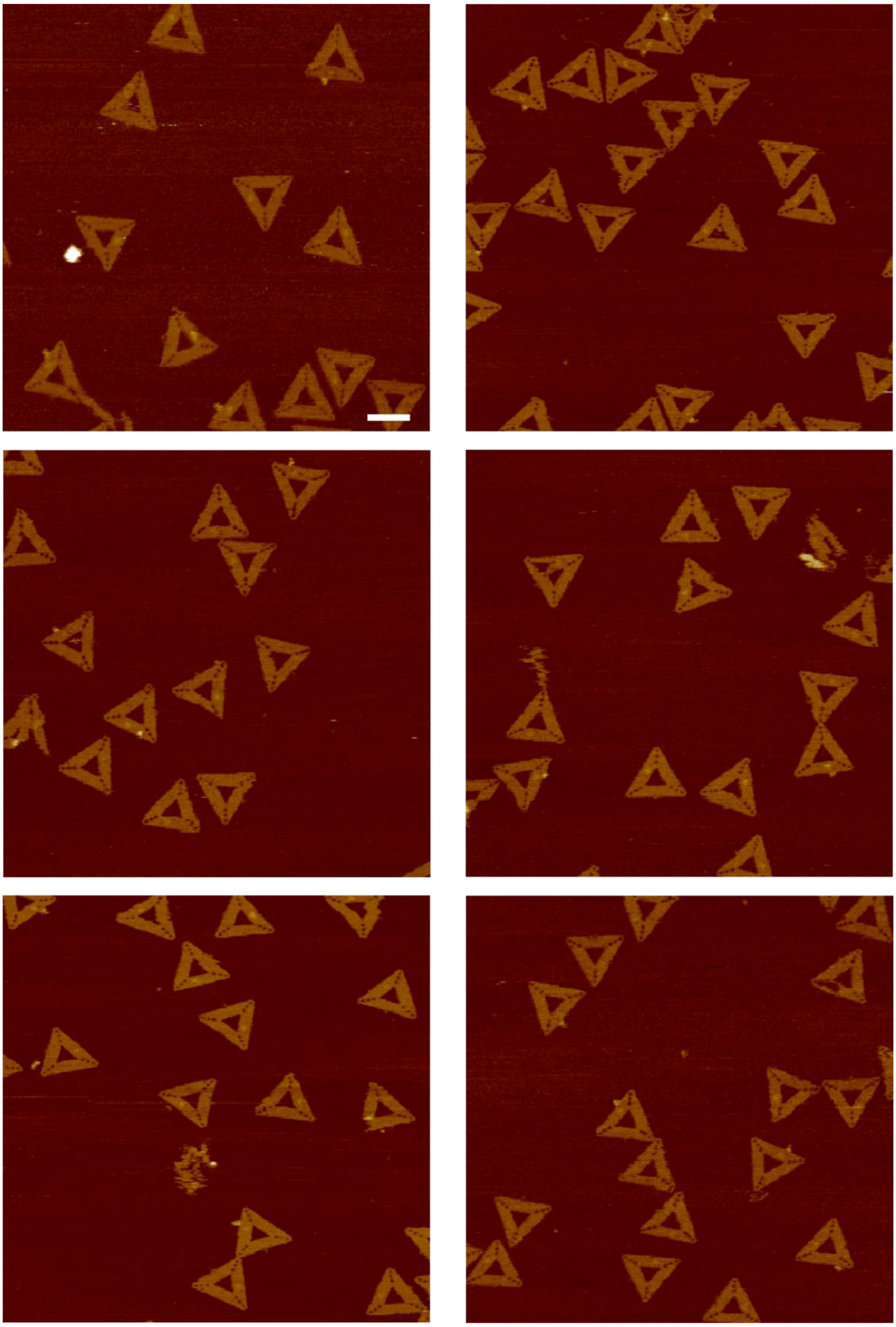

Supplementary Figure 29. PeakForce-AFM images of Cholesterol-IgG complexes in a final IgG concentration of 78 nM. Scale bar, 100 nm.

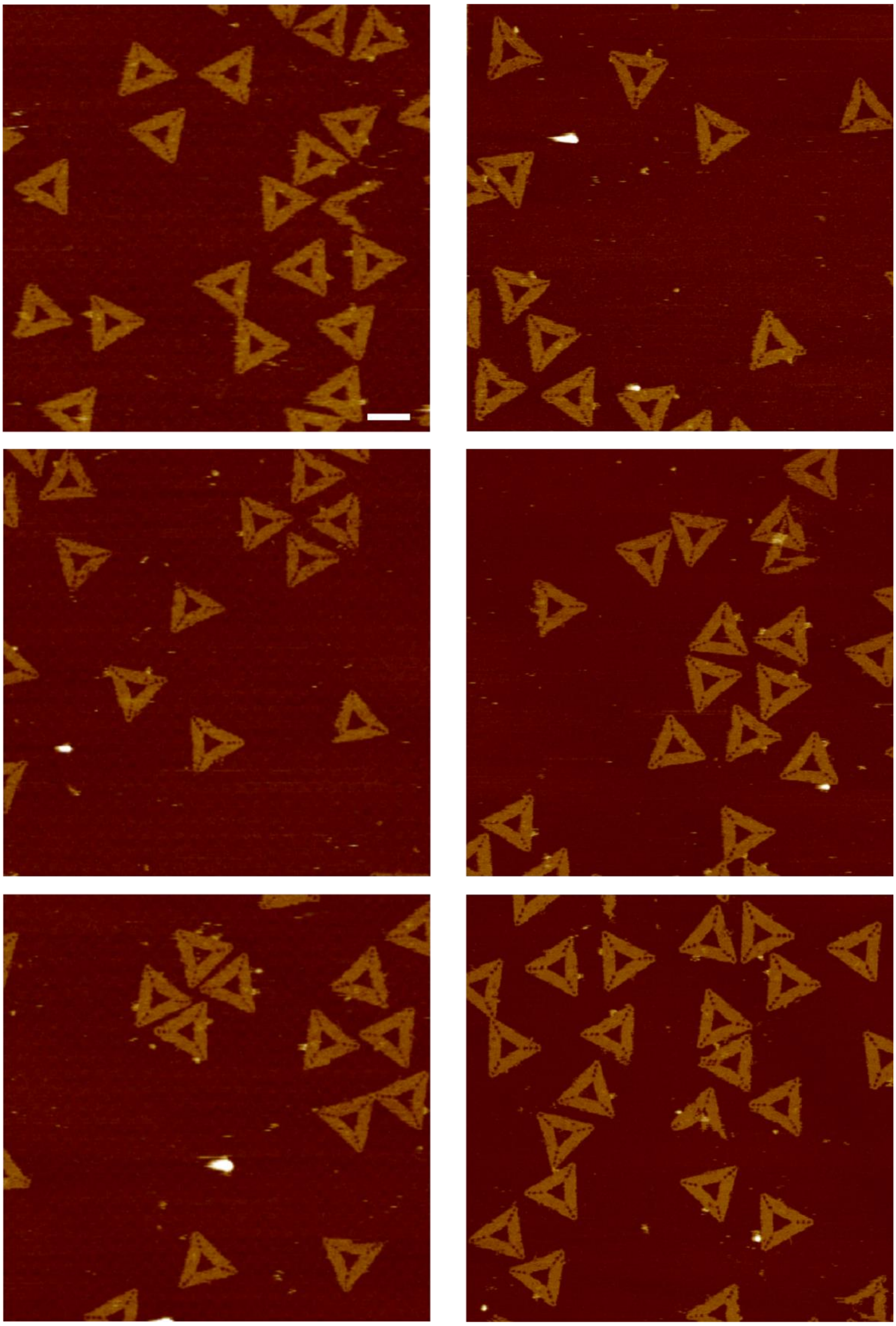

Supplementary Figure 30. PeakForce-AFM images of Cholesterol-IgG complexes in a final IgG concentration of 156 nM. Scale bar, 100 nm.

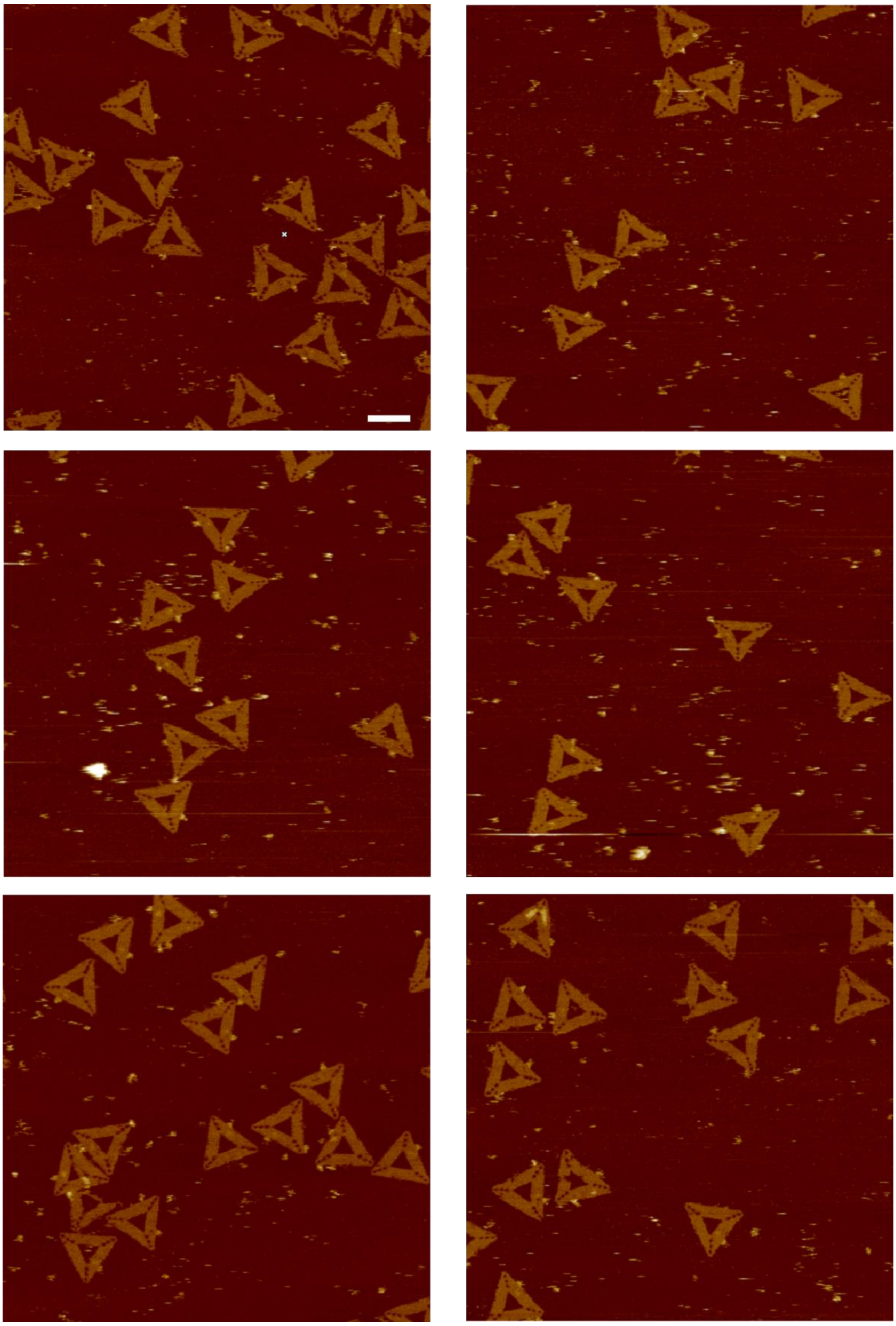

Supplementary Figure 31. PeakForce-AFM images of Cholesterol-IgG complexes in a final IgG concentration of 312 nM. Scale bar, 100 nm.

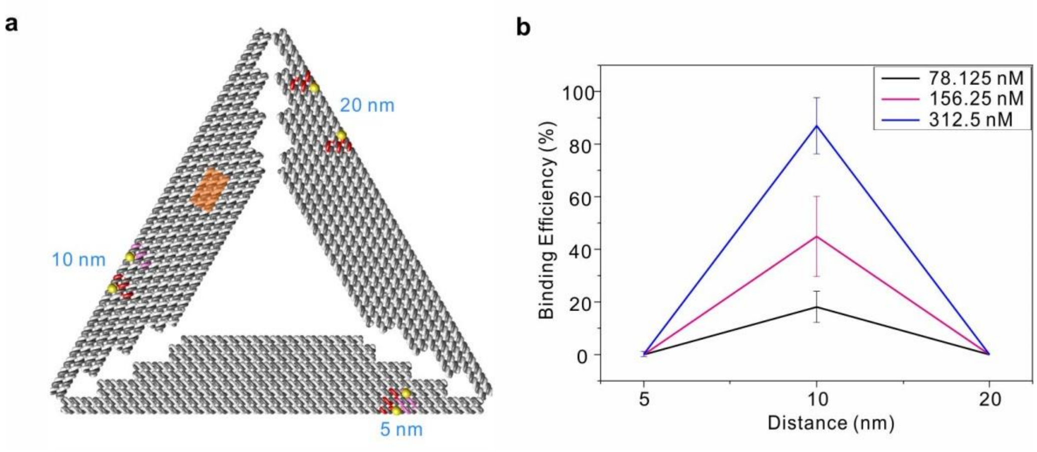

Supplementary Figure 32. Binding efficiency of cholesterol-IgG complexes a, Schematic diagram for the location of various designed distances. **b,** Binding efficiency of IgGs as a function of lateral distance of cholesterol using various concentrations of IgGs. The error bars represent the standard deviation, which was calculated from three independent experiments for each concentration of IgGs, n = 661 DNA origamis**.** Source data are provided as a Source Data file.
