# Supplementary material for: Capturing transient antibody conformations with DNA origami epitopes
Source: Nat Commun. 2020 Jun 19;11:3114. doi: 10.1038/s41467-020-16949-4 (PMC7305102; doi:10.1038/s41467-020-16949-4)
Supplement: Supplementary file 2 — Description of Additional Supplementary Files [file 41467_2020_16949_MOESM2_ESM.docx]

Description of Additional Supplementary Files

**Title: Supplementary Movie 1**

Description: Dynamics of IgG binding to lateral digoxin distance 3 nm

**Title: Supplementary Movie 2**

Description: Dynamics of IgG binding to lateral digoxin distance 5 nm

**Title: Supplementary Movie 3**

Description: Dynamics of IgG binding to lateral digoxin distance 8 nm

**Title: Supplementary Movie 4**

Description: Dynamics of IgG binding to lateral digoxin distance 10 nm

**Title: Supplementary Movie 5**

Description: Dynamics of IgG binding to lateral digoxin distance 16 nm
